# Benchmarking mobile applications for the health of people with Diabetes Mellitus ^*^

**DOI:** 10.1590/1518-8345.7182.4221

**Published:** 2024-07-05

**Authors:** Rafael Oliveira Pitta Lopes, Sara Rodrigues Chagas, Eduardo da Silva Gomes, Joice Cesar de Aguiar Barbosa, Ítalo Rodolfo Silva, Marcos Antônio Gomes Brandão

**Affiliations:** ^1^ Universidade Federal do Rio de Janeiro, Escola de Enfermagem Anna Nery, Rio de Janeiro, RJ, Brazil.; ^2^ Universidade Federal do Rio de Janeiro, Instituto de Enfermagem do Centro Multidisciplinar, Macaé, RJ, Brazil.; ^3^ São Lucas Hospital de Clínicas, Macaé, Rio de Janeiro, RJ, Brazil.; ^4^ Scholarship holder at the Coordenação de Aperfeiçoamento de Pessoal de Nível Superior (CAPES), Brazil.; ^5^ Scholarship holder at the Conselho Nacional de Desenvolvimento Científico e Tecnológico (CNPq), Brazil.

**Keywords:** Diabetes Mellitus, Mobile Applications, eHealth Strategies, Telenursing, Health Promotion, Digital Inclusion

## Abstract

**Objective:**

to map the content and features of mobile applications on the management of Diabetes Mellitus and their usability on the main operating systems.

**Method:**

benchmarking research. The mapping of apps, content, and resources on the Play Store and App Store platforms was based on an adaptation of the Joanna Briggs Institute’s scoping review framework. For the usability analysis, the apps were tested for two weeks and the System Usability Scale instrument was used, with scores between 50-67 points being considered borderline, between 68-84, products with acceptable usability and above 85, excellent user acceptance and, for the analysis, descriptive statistics.

**Results:**

the most prevalent contents were capillary blood glucose management, diet, oral drug therapy, and insulin therapy. As for resources, diaries and graphs were the most common. With regard to usability, two apps were considered to have excellent usability; 34, products with acceptable usability; 29, the resource may have some flaws but still has acceptable usability standards and 6, with flaws and no usability conditions.

**Conclusion:**

the content and resources of mobile applications address the fundamental points for managing Diabetes Mellitus with user-friendly resources, with usability acceptable to users and have the potential to assist in the management of Diabetes Mellitus in patients’ daily lives.

## Highlights

**(1)** The usability of digital platform apps was systematically evaluated.

**(2)** Most apps for people with Diabetes Mellitus have good usability.

**(3)** The study provides an overview of the features available in the apps.

**(4)** The apps have the potential to help in the management of Diabetes Mellitus.

**(5)** The apps should be evaluated by reliability criteria for recommendation.

## Introduction

 Diabetes Mellitus (DM) can lead to serious health complications in all age groups, including young people, and these complications are the main reasons for the worsening quality of life, disability, and death of people with DM ^(^ ^1^ ^-^ ^2^ ^)^ . 

 The health management of people with DM should prioritize the establishment of a healthy lifestyle with habits and practices that favor well-being and coping with the challenges of everyday life ^(^ ^3^ ^)^ . Such management requires multiple responsibilities on the part of the person in terms of self-management of their daily life, awareness of self-concept, development of autonomy, management of social relationships and roles in the work environment, affective relationships and emotional responses, and support; as well as others that require strong professional monitoring, such as those related to safety and adherence to drug treatment, variations in blood glucose and any biological or drug responses ^(^ ^3^ ^)^ . 

 There are indications in the literature that the use of technologies for DM improves health outcomes by reducing barriers to accessing health guidance, especially through mobile devices; with free access and centralization of functions in just one device that can promote autonomy and facilitate disease management. In addition, these technologies can make a positive contribution to decision-making on blood glucose monitoring and management, automating everyday processes or improving the solution to health-related problems ^(^ ^4^ ^-^ ^7^ ^)^ . 

 Although there are advantages to using mobile devices, there is also evidence of barriers to the use of apps among people with DM. These barriers may include poor usability, the lack of accessibility features, and the content present in the apps, thus constituting obstacles for users ^(^ ^8^ ^)^ . In addition, both in professional clinical practice and in users’ daily lives, the identification of these apps is not systematized. The selection of these resources must be supported by an efficient analysis of content, resources, and usability, ensuring the safe use of these tools for health management. In this way, technology performance evaluation studies are used to analyze and evaluate technologies and thus provide elements for interpreting the benefits and limitations of the technology ^(^ ^9^ ^-^ ^11^ ^)^ . 

 A systematic review published in 2017 on the usability and clinical effectiveness of mobile apps for adults with Type 2 Diabetes Mellitus (DM2) showed that twenty publications on usability, user satisfaction, and usage problems indicated limited effectiveness of mobile apps in promoting glycemic control ^(^ ^12^ ^)^ . In 2019, a study presented the results of the identification of apps available in Spanish on the Play Store (Android) and App Store-iPhone Operating System (iOS) platforms, indicating thirty-four apps, of which three did not have quality certification and only three indicated the scientific references used ^(^ ^13^ ^)^ . 

In view of the above, challenges remain in identifying available mobile applications for managing DM, as well as verifying their usability properties. Therefore, the aim of this article is to map the content and features of mobile applications for managing DM and their usability on the main operating systems.

## Methods

### Study design

 This is a benchmarking study ^(^ ^10^ ^-^ ^11^ ^)^ of mobile applications, which sought, by mapping application platforms, to survey the content, resources, and usability of mobile applications aimed at the health of people with DM. 

 A benchmarking study makes it possible to evaluate the performance and results of a resource or technology, making comparisons with other resources, with a view to providing improvements and innovative ideas. It acts as a search tool that allows for the improvement and modern design of apps with the best evidence and functionalities ^(^ ^10^ ^-^ ^11^ ^)^ . 

The scoping review framework, according to the Joanna Briggs Institute, was adapted to map apps and synthesize their content and resources. The adapted methodological steps were: 1) Definition and alignment of the objective and question; 2) Development and alignment of the inclusion criteria with the objective and question; 3) Description of the approach planned for the search, selection, extraction of content, and resources, and presentation of the apps; 4) Search for apps; 5) Selection of apps; 6) Extraction of content and resources; 7) Analysis of content, resources, and usability; 8) Presentation of the results and, 9) Summary in relation to the purpose, conclusions and implications of the conclusions.

The aim was to identify mobile apps aimed at the health of people with DM, their content, resources, and usability. To this end, the question was developed: “What are the contents, resources, and usability of apps aimed at the health of people with DM?”. In order to achieve the objective, the inclusion criteria were: apps in Portuguese, English, and Spanish, which included the theme of health and DM and those which: 1) required payment for installation; 2) were aimed at health professionals; 3) required permission from the authors for their use (registration number and password); 4) were aimed at accompanying a scientific event/conference; 5) were games and knowledge evaluators; 6) were aimed at animal health; 7) were unavailable for download or use; 8) required specific sensors for their operation. The search approach was guided by the terms “diabetes mellitus” and “health”.

The search, selection, and download were carried out between November and December 2021, in the Play Store and App Store virtual stores, of the Android (Google) and iOS (Apple) operating systems, respectively. Two researchers independently searched for and downloaded the apps from the online stores. The first researcher used an Android-compatible Samsung Galaxy A71 and an iOS-compatible iPhone 6s. The second researcher used a Xiaomi 8 Lite, compatible with Android, and an iPhone 6s, compatible with iOS. Four searches were carried out in each online store, using each of the previously established terms individually. After the search, the apps were selected via title and short description. The apps that met the inclusion and exclusion criteria were downloaded and analyzed.

 To collect and organize the information extracted, each researcher organized the app data into an information matrix in Microsoft Excel ^®^ , with a description of identification (name, developer, author(s) responsible, year of release, language, country of origin); target audience (patient, family members/caregivers and students); app data (description, current version, number of downloads, rating/evaluation). 

Two researchers then analyzed the usability of each app in tests that lasted two weeks. This evaluation period was necessary so that some apps could produce a minimum set of data capable of generating user graphics. At the end of this period, the researchers had enough elements to reliably analyze usability.

### Usability data analysis

 The usability analysis of the apps was based on the System Usability Scale (SUS) questionnaire, which was validated for use in Brazil in 2010 ^(^ ^14^ ^)^ . The SUS evaluates products, software, applications, interfaces, and websites by criteria of efficiency, effectiveness, and satisfaction, and allows the identification of advantages and weaknesses through the previous use of the selected apps. The questionnaire has ten items that show a comprehensive view of the user in relation to the system, with the user having to select an item from a scale ranging from 1 (Completely Disagree) to 5 (Completely Agree) ^(^ ^14^ ^)^ . 

 To calculate usability, 1 was subtracted from the score for odd answers and 5 was subtracted for even answers. To obtain the final average, the value found was multiplied by 2.5, which produced the final score (between 0 and 100). In relation to the SUS scores, a score between 50-67 points is considered borderline, i.e., the resource may have some flaws, but still has acceptable usability standards. Scores between 68-84 represent products with acceptable usability. Software that scores above 85 has excellent user acceptance ^(^ ^15^ ^)^ . After the independent evaluation, the two researchers compared the results of the SUS application for each app, discussing the possible differences in scores for the ten items in the questionnaire. This debate was mediated by the concomitant review of the app by the two researchers until a consensus was reached to determine the app’s final usability score. 

### Ethical aspects

This study was not submitted to a Research Ethics Committee because it was characterized as research that did not directly involve human beings, whose data is in the public domain, and did not involve any commercial, professional, or other conflicts of interest. The information collected from the apps was used exclusively for scientific purposes. The apps analyzed were duly mentioned in the study.

## Results

**Figure 1:**
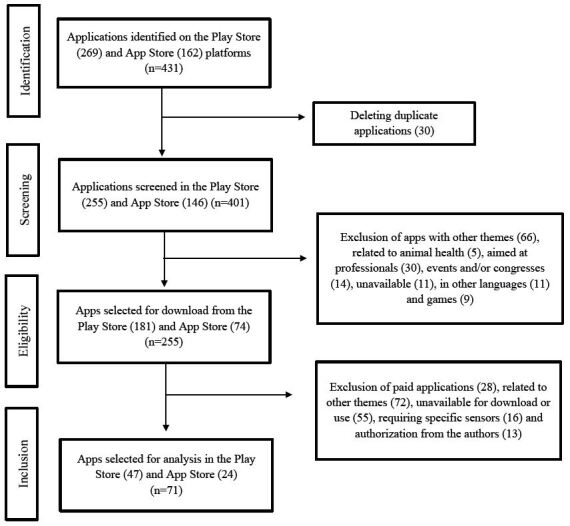
- Flow diagram of the selection of applications on the selected virtual platforms. Macaé, RJ, 2023

 431 apps were identified on the app platforms, 269 on the Play Store, and 162 on the App Store. Of these, 30 were excluded for being duplicates, leaving 401 apps. After analyzing the titles and describing the content, 255 apps were selected for download and detailed evaluation. In the end, the sample consisted of 71 apps. The app selection process is described in Figure 1 . 

The apps analyzed have been available on the platforms since 2010, with an increase in the number of developments since 2013. It was observed that 2020 was the period with the highest number of launches, totaling 12 (16.9%) apps. However, a decline in app development was identified after 2021.

The majority of apps had developers from the United States (21; 29.5%) and India (14; 19.7%). This was followed by apps from Brazil (6; 8.4%); France (4; 5.6%); Germany (3; 4.2%); Pakistan, Spain, Russia, Canada and China (2; 2.8% each); Austria, Greece, Poland, Switzerland, Turkey, the United Kingdom, the Netherlands, Bulgaria, Denmark, Japan, Italy, Finland and Belgium (1; 1.4% each).

 DM management was covered in all the apps, involving content such as self-monitoring, control, and management of glycemic patterns, recording of physical activities, educational guidelines related to diet, information on the use of medication and insulin, as well as pressure control and anthropometric measurements ( Figure 2 ). 

**Figure 2:** - Characterization of apps according to language, objectives, version, evaluation, and downloads. Macaé, RJ, 2023

**Application**	**Language**	**Description/Objective**	**Version**	**Stars rating**	**Downloads**
mySugr	Portuguese	Manage DM ^*^ through daily records	3.92.10	4.5	>1,000,000
Diabetes: Management & Blood Sugar Tracker App ^†^	Portuguese	Monitor, analyze, and keep DM ^*^ under control	8.0.11	4.4	>500,000
Blood Sugar Diary	English	Blood glucose diary	1.1.1	4.0	>500,000
Blood Glucose Tracker	English	Recording blood glucose levels	1.8.12	4.7	>500,000
*Diabete – Diário Glucose*	English	Blood glucose diary	4.4	4.6	>500,000
*A Minha Glycemia*	Portuguese	Glucose monitoring tool	1.8.2	4.2	>100,000
BD ^‡^ Diabetes Care	Portuguese	Managing DM ^*^	3.1.2	3,3	>100,000
Beat Diabetes	English	Recipes and diet tips	9.9	4.7	>100,000
Blood Sugar Log – Diabetes Tracker	English	Track blood glucose, BP ^§^ , weight, and medication records	1.13	4.5	>100,000
*Controle de Glicose*	Spanish	Blood glucose diary	2.0.3	4.1	>100,000
*Diabéticos Receitas*	Portuguese	Healthy recipes tool	45.0.0	4.1	>100,000
Diabetes Diary – Blood Glucose Tracker	English	Track glucose levels, BP ^§^ , weight, and HbA1c ^||^	1.26	4.7	>100,000
*Diário Diabetes*	English	Blood glucose and HbA1c ^||^ diary	2.2.9	3.9	>100,000
*Intellin Diabetes Log Tracker & Risk Manager App* ^†^	English	Manage DM ^*^	1.10.167	4.0	>100,000
Social Diabetes	Portuguese	Better control through data records	4.17.53	4.0	>100,000
BG ^¶^ Monitor Diabetes	English	Manage insulin use and physical exercise	8.0.1	4.3	>50,000
*Diabetes Controle*	Portuguese	Insulin diary	1.5	4.6	>50,000
Diabetes Tracker Free version	English	Blood glucose diary	1.9	3.9	>50,000
Glycemic Index	English	Food glycemic index	2.15.17	4.5	>50,000
Blood Glucose Tracker	English	Managing DM ^*^	2.0	4.3	>10,000
Diabetes Control App ^†^	English	Storing and managing DM control data ^*^	3.4.1.0	4.1	>10,000
*Diabetes Ginástica*	English	Exercise routines for diabetic users	1.3	3.9	>10,000
Diabetes Logbook – Blood Glucose Tracker	English	Keeping a blood glucose diary and counting carbohydrates	2.7	3.8	>10,000
Diabetic Recipes: Great Recipes for Diabetics	English	Recipes for diabetics	2.0	4.7	>10,000
*Diário de Sangue*	Portuguese	Blood glucose diary	2.2.0	3.9	>10,000
forDiabetes: *Aplicativo de Diabetes*	Variety	Record and monitor essential data for DM ^*^ control	1.119.4	4.5	>10,000
*Glucômetro – Rastreamento de Diabetes*	English	Track glucose levels, HbA1c ^||^ and insulin administered	2.16	4.2	>10,000
Glucool Diabetes	English	Managing DM ^*^	1.4.3.1	3.7	>10,000
My Sugar Diary – Diabetes App ^†^	English	Record glucose levels	1.8.6	4.2	>10.000
Blood Glucose Tracker – Track Your Blood Glucose	English	Manage DM ^*^ by recording glucose levels, weight, and BP ^§^	1.9.2	4.2	>5,000
Diabetes Food Recipes	English	Recipes for diabetics	3.1.4	4.2	>1,000
Sukar – Blood Glucose & Diabetes Tracker App ^†^	English	Managing DM ^*^	1.1.6	3.7	>1,000
Diabetes Test My Blood Sugar Diary – BP ^§^ Glucose	English	Manage glucose levels, HbA1c ^||^ , weight and BP ^§^ in the form of statistics	04.04.2021	4.8	>1,000
Wecheck – Diabetes Lifelog	English	Manage DM ^*^	1.0.9	4.1	>1,000
Blue Circle Diabetes	English	Manage DM ^*^	1.25	4.8	>1,000
Diabetes Tracker App: Blood Glucose & Cholesterol	English	Record and track glucose and cholesterol levels	1.0	3.9	>1,000
Diabetes Tracker	English	Manage DM ^*^	1.0	4.0	>1,000
Joddi – Diabetes LogBook	English	Diary for blood glucose control	0.7.2	4.1	>1,000
Eglucomonitor – Sugar & Diabetes Monitor App ^†^	English	Daily blood glucose monitoring	1.0.1	NI ^**^	>500
Blood Sugar Diary - Blood Glucose Tracker	English	Blood glucose diary	1.2	4.8	>100
Butterfly – Diabetes App ^†^	English	Tool with weekly healthy goals for DM ^*^ control	10.0	NI ^**^	>100
Diabetic Diet Plan	English	Recipes for diabetics	1.3	NI ^**^	>100
Diabetes Meal Plan	English	Meal plan for people with DM ^*^	1.0	NI ^**^	>100
*Receitas para diabéticos*	Portuguese	Food and recipes for diabetics	1.00	4.9	>100
Diabetic Diet Plan	English	Food plan for diabetics	1.0	NI ^**^	>50
Smart Diabetes Diary	English	Record blood glucose, insulin, and medication values	1.0	NI ^**^	>10
Carbok: Carb Counting & Management for Diabetics	English	Carbohydrate counting	2.1.0	NI ^**^	>5
Blood Sugar Diary for Diabetes	English	Blood glucose diary	2.11	NI ^**^	NI ^**^
Center Health – The Diabetes App ^†^	English	Blood glucose diary	3.4	NI ^**^	NI ^**^
Contagem de Carboidratos	Portuguese	Carbohydrate counting	2.1	NI ^**^	NI ^**^
DiabetesDocs	English	Tool for managing DM ^*^	4.6	NI ^**^	NI ^**^
*Diabética Comida Receitas App* ^†^	Portuguese	Healthy recipes for diabetics	4.1	NI ^**^	NI ^**^
Diabetes Diary	English	Tool for controlling blood glucose, insulin and carbohydrate intake	2.1	NI ^**^	NI ^**^
Diabetes Health Manager	English	Managing DM ^*^	10.7	NI ^**^	NI ^**^
Diabetes Pilot Pro	English	Blood glucose diary	7.0	NI ^**^	NI ^**^
Diabetes Pro	English	Managing DM ^*^	1.5	NI ^**^	NI ^**^
Diabetes SmartManager	English	Tool for calculating insulin doses	1.1	NI ^**^	NI ^**^
Dits	English	Food and blood glucose diary tool with carbohydrate counting	1.4	NI ^**^	NI ^**^
*Glic Diabetes e Glicemia*	Portuguese	Blood glucose diary	4.1	NI ^**^	NI ^**^
*GlicAPP*	Portuguese	Blood glucose recording and measurement reminder tool	1.3	5.0	NI ^**^
*Glicose Companheiro*	English	Blood glucose diary	7.2	NI ^**^	NI ^**^
glucoSecrets	English	Blood glucose diary	5.2	NI ^**^	NI ^**^
GlucoseMonitor	English	Tool for controlling blood glucose, weight, and medication	3.10	4.5	NI ^**^
gluQUO: Control Your Diabetes	English	Tool for managing DM ^*^	2.3	NI ^**^	NI ^**^
Happy Bob	English	Tool with encouraging advice for controlling DM ^*^	1.9	NI ^**^	NI ^**^
Health2Sync	English	Blood glucose diary	2.6	NI ^**^	NI ^**^
Help Diabetes	English	Carbohydrate counting tool	3.1	NI ^**^	NI ^**^
iGlicho	Portuguese	Managing DM ^*^	0.8	NI ^**^	NI ^**^
Insulin Calculator	Portuguese	Calculate insulin doses	1.0	NI ^**^	NI ^**^
*Diabetic patients, follow up and monitor their blood glucose levels*	Portuguese	Managing DM ^*^	1.6	NI ^**^	NI ^**^
Undermyfork: Diabetes	English	Food diary for people with DM ^*^	2.12	NI ^**^	NI ^**^

^*^ DM = *Diabetes Mellitus* ;

^†^ *App* = Application;

^‡^ BD = *Becton Dickinson* ;

^§^ BP = Blood Pressure;

^||^ HbA1c = Glycated Hemoglobin;

^¶^ BG = Blood Glucose;

^**^ NI = Not Identified

Three thematic axes can represent the content and resources that indicate the central purpose of the apps: “apps for managing blood glucose and drug therapy”, encompassing actions to manage the pathophysiology and treatment of DM; “apps for lifestyle changes”, such as physical activity, diet, blood pressure, and weight control; and “apps for well-being and education measures”, with goal-setting, motivational guidance, encouragement to live with the chronicity of DM and educational guidance.

**Figure 3:**
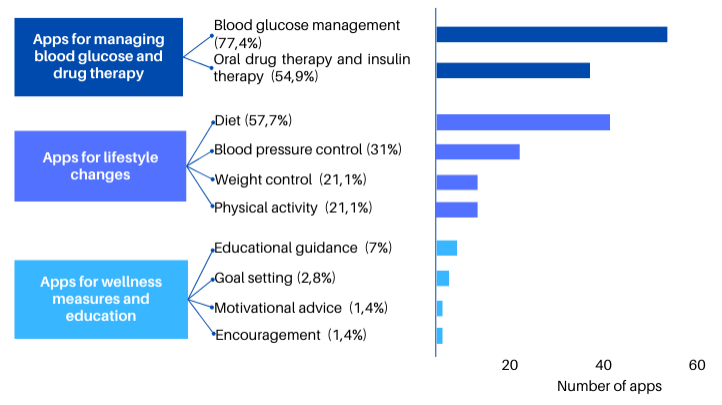
- Categorization of apps according to the thematic axes and topics covered. Macaé RJ, 2023

Figure 3 shows the categorization of the apps analyzed in terms of the thematic axes and the topics covered. The predominant themes were blood glucose management (55; 77.4%), followed by diet (41; 57.7%), oral drug therapy, and insulin therapy (39; 54.9%). Some apps include more than one theme in their content, allowing them to fit into one or more categories at the same time. 

With regard to the resources available on the apps, the following were identified: diaries (50; 70.4%); graphs (46; 64.7%); figures/images (37; 52.1%); reports (27; 38%); reminders/alarms (17; 23.9%), statistics (7; 9.8%) and tables (5; 7%). Features related to lifestyle and well-being were also identified: recording blood pressure (22; 30.9%), weight (14; 19.7%), monitoring physical activity (15; 21.1%) and guidelines/goals (2; 2.8%) which help in the effective management of DM. Some apps allowed data to be entered on the user’s sociodemographic profile (47; 66.2%) and clinical profile (52; 73.2%), such as the year of diagnosis (9; 12.6%) and the type of DM (24; 33.8%).

 With regard to the usability of the apps analyzed, two (2.8%) obtained a score ≥ 85; 34 (47.9%) scored between 68-84 points; 29 (40.8%) had scores between 50-67 points; six (8.5%) obtained scores below 50. Figure 4 illustrates the apps’ information on content, features, and usability. 

**Figure 4:** - Characterization of the selected apps in terms of content, resources and usability. Macaé, RJ, 2023

**Application**	**Content**	**Features**	**Usability** (SUS ^*^ points)
Excellent usability (n=2)			
forDiabetes: *Aplicativo de diabetes*	Blood glucose, medications, BP ^†^ , meals, physical activity, weight, HbA1c ^‡^ , and articles about DM ^§^ .	Texts, pictures, graphs, reminders, report sharing, synchronization with other devices; enter daily activities, meals, medications, blood glucose levels, BP ^†^ , HbA1c ^‡^ , and weight.	85.0
Carbok: Carb Couting & Management for Diabetic	Diet and carbohydrate counting.	Figures, texts, calculator, and graphs; it allows you to record the characteristics of meals.	85.0
Acceptable usability (n=37)			
mySugr	Blood glucose diary, medication management, insulin and carbohydrate intake.	Text, graphics, and pictures. You can add reminders, record blood glucose, steps/physical activity, insulin/medications, and weight, and send reports.	82.5
Diabetes: Management & Blood Sugar Tracker App ^||^	Recording blood glucose, medication management, food, weight, BP ^†^ , physical activity, and laboratory tests.	Texts, graphs, and figures. It allows you to record blood glucose levels, food, and medication, set alarms and monitor progress using graphs and reports.	82.5
*A minha glycemia*	Capillary blood glucose, insulin, HbA1c ^‡^ , and BP ^†^ .	Enter blood glucose values, reminders, tables, histograms, notepad, and add contacts.	82.5
Blood Glucose Tracker	Food, weight, BP ^†^ , blood glucose and medication.	Texts, graphs, pictures, and notebooks.	82.5
Center Health – The Diabetes App ^||^	Diary of blood glucose, insulin, carbohydrate counting, exercise, and sleep.	Texts, graphs, figures, data logging, sensor synchronization, spreadsheet import and export.	82.5
*Contagem de Carboidratos*	Carbohydrate counting.	Graphs, texts, and calculator.	82.5
*Glic Diabetes e Glicemia*	Blood glucose, insulin, BP ^†^ , medicines, and educational articles.	Figures, texts, graphs, hypoglycemia alerts, nutritional tables, insulin calculation, carbohydrate counting, connect the electronic medical record, and send the data report.	82.5
iGlicho	Diet, blood glucose diary, carbohydrate counting, and insulin.	Graphs, texts, food tables, carbohydrate counting, insulin calculation, and report generation.	82.5
*Controle de Glicose*	Blood glucose, HbA1c ^‡^ , insulin, medication, BP ^†^ , and diet.	Graphs, texts. It allows you to add laboratory tests, and view glucose, and BP ^†^ levels in graphs and export reports.	80.0
Smart Diabetes Diary	Blood glucose, insulin, and medication.	Figures, graphs, statistics, texts, notepad, and data export.	80.0
Diabetes Logbook – Blood Glucose Tracker	Diary of blood glucose, HbA1c ^‡^ , carbohydrate count, and insulin.	Texts, graphs, and figures.	80.0
*Diabética Comida Receitas App|* ^|^	Diet.	Texts and images.	80.0
Health2Sync	Diary of blood glucose, diet, carbohydrate counting, physical activity, BP ^†^ , and weight.	Graphs, text, pictures. You can add photos of your meals and generate reports.	80.0
Dits	Capillary blood glucose and educational articles.	Graphs, texts, figures, and articles.	80.0
Blood Sugar Log – Diabetes Tracker	BP ^†^ , blood glucose, Hb1Ac ^‡^ , weight and medication.	Figures, texts, and sending reports.	77.5
Diabetes Meal Plan	Diet.	Images, texts, and calculator	77.5
Blood Glucose Tracker	Blood glucose, BP ^†^ , medication, Hb1Ac ^‡^ , and weight.	Graphs, statistics, texts, and notepad.	77.5
Diabetes Tracker App ^||^ : Blood Glucose & Cholesterol	BP ^†^ , weight, blood glucose, HbA1c ^‡^ , and cholesterol.	Texts, reminders, and graphics.	77.5
Diabetes Tracker	Physical activity, diet, medication, BP ^†^ , and blood glucose.	Pictures, reminders, voice search, add a list of professionals, date of appointments, and reports.	77.5
*Glicose Companheiro*	Blood sugar and weight.	Graphs, statistics, texts, calculator, and reports.	77.5
Happy Bob	Glycemic self-monitoring, goals, and advice for controlling DM ^§^ .	Texts, figures.	77.5
Blood Sugar Diary for Diabetes	Automated glucose monitoring.	Texts, statistics, notifications, graphs, theme changes, articles, and synchronization with sensors.	77.5
*SocialDiabetes*	Vital signs, blood glucose diary, HbA1c ^‡^ , medication, diet, physical activity, encouragement, and educational content.	Texts, calculator, HbA1c ^‡^ estimation, alarms, sensor synchronization, and report export.	75.0
Diabetes Health Manager	Symptoms, medication, treatment, and glycemic monitoring.	Figures, texts, graphs, and articles.	75.0
*Diabéticos Receitas*	Healthy recipes.	Healthy recipes. Texts and pictures. It allows you to favorite recipes, create shopping lists, and search for recipes by selecting a specific ingredient/food.	72.5
*Blue Circle Diabetes*	Physical activity, diet, insulin, and blood glucose.	Texts, images, calculator, online store, nutritional information, recipes, and online meetings about DM ^§^ .	72.5
Eglucomonitor – Sugar & Diabetes Monitor App ^||^	Blood glucose and insulin diary.	Texts and graphics.	72.5
Diabetes Diary	Blood glucose, carbohydrate counting, and insulin.	Graphs, texts, and calculator.	72.5
BD ^¶^ Diabetes Care	Blood glucose and insulin recording; healthy recipes, questions and answers about DM ^§^ and educational content.	Texts and pictures. Allows you to record blood glucose and insulin, share reports, and reminders, connect to other devices, questions and answers, search for recipes and educational content, and set daily goals.	70.0
Diabetes Diary – Blood Glucose Tracker	BP ^†^ , weight, HbA1c ^‡^ , medication and blood glucose.	Texts, graphs, and reports.	70.0
Intellin Diabetes Log Tracker & Risk Manager App	Physical activity, BP ^†^ , blood glucose, and insulin.	Texts, figures, graphs, synchronization with other devices, and report sharing.	70.0
Glycemic Index	Nutrition.	Texts and tools for adding new foods.	70.0
Undermyfork: Diabetes	Diet and glycemic diary.	Texts, images, and graphics.	70.0
Insulin Calculator	Insulin.	Texts and graphics. It allows you to create meals, calculate insulin, and obtain history.	70.0
*BG* ^**^ *Monitor Diabetes*	Diary of blood glucose, insulin, physical activity, and diet.	Graphs and text. It has reminders and reports.	67.5
*Diabetes Tracker Gratuito*	Glycemic diary.	Text, graphics, and reminders.	67.5
*Receitas para Diabéticos*	Nutrition.	Images and texts.	67.5
Limited usability (n=26)			
Diabetes Control App ^||^	Food, insulin, medication, and blood glucose.	Texts and graphics. Reminder, notepad, add professional contact, chat, and reports.	65.0
Diabetes Controle	Glycemic self-monitoring.	Graphs, statistics, figures, texts, and share reports.	65.0
Blood Glucose Tracker – Track Your Blood Glucose	Glycemic diary, BP ^†^ , and weight.	Texts, graphs, and export data.	65.0
*Glucômetro – Rastreamento de Diabetes*	Diary of blood glucose, insulin, and HbA1c ^‡^ .	Enter glucose, HbA1c ^‡^ , and insulin levels.	65.0
Diabetes Test My Blood Sugar Diary – BP ^†^ Glucose	Self-monitoring of blood glucose, BP ^†^ , weight, HbA1c ^‡^ , and medication.	Figures, texts, graphs, and export reports.	65.0
Blood Sugar Diary - Blood Glucose Tracker	Food, medication, and blood glucose.	Texts, figures, graphs, and articles about DM ^§^ .	65.0
GlucoseMonitor	Glycemic diary and vital signs.	Graphs, texts, and reminders.	65.0
gluQUO: Control Your Diabetes	Diet, physical activity, and blood glucose diary.	Texts, pictures, graphs, reminders, calculator; allows you to add photos of meals and get reports to share.	65.0
GlicAPP	Laboratory tests, blood glucose, and insulin.	Texts. Allows you to record medical data, laboratory tests, and suggestions.	65.0
My Sugar Diary – Diabetes App ^||^	Blood glucose diary.	Texts, tables, and figures	62.5
Diabetes Pro	Blood glucose diary and medication.	Figures, texts, graphs, and a tool to rotate data for checking capillary glycemia.	62.5
Diabetes Food Recipes	Diet.	Texts and images.	60.0
Diabetes Pilot Pro	Blood glucose diary, diet, and carbohydrate counting.	Texts and graphics.	60.0
Diabetic patients, follow up and monitor their blood glucose levels	Blood glucose diary.	Texts, tables, graphs, and reminders.	60.0
*Diário Diabetes*	Physical activity, diet, BP ^†^ , weight, glucose, HbA1c ^‡^ , and medication.	Texts, graphs, averages of entered data, and reminders.	57.5
Glucool Diabetes	Blood glucose, insulin, BP ^†^ , carbohydrate counting, HbA1c ^‡^ , exercise, and medication.	Graphs, texts, calculator, and data export.	57.5
Joddi – Diabetes LogBook	Blood glucose and insulin diary.	Texts, recording, and exporting data.	57.5
Diabetic Diet Plan (v. 1.0)	Diet.	Texts and images.	57.5
Diabetic Diet Plan (v. 1.3)	Diet.	Images and texts.	57.5
glucoSecrets	Blood glucose diary, physical activity, and medication.	Graphs/statistics, texts, and figures.	57.5
Help Diabetes	Carbohydrate counting.	Texts and calculator.	57.5
Beat Diabetes	Diet.	Images and texts.	52.5
*Diabete – Diário Glucose*	Blood glucose and weight.	Graphs, statistics, reminders, entering values, exporting and importing data.	52.5
Wecheck – Diabetes Lifelog	Vital signs, diet, insulin and blood glucose.	Graphs, texts, recording of vital signs, glucose and insulin; NFC ^††^ connection, Bluetooth and synchronization with Wecheck devices.	52.5
Butterfly – Diabetes App ^||^	Weekly targets for DM ^§^ control.	Texts and data recording.	52.5
Diabetes SmartManager	Carbohydrate counting, insulin and blood glucose diary.	Texts, reminders, graphs. Allows you to record blood glucose and insulin, change the color of the theme and obtain reports.	50.0
Inadequate usability (n=6)			
*Diário de Sangue*	Glycemic diary, BP ^†^ , and weight.	Charts, calendar, and recording of blood glucose, BP ^†^ , and weight.	47.5
Blood Sugar Diary	Blood glucose diary.	Texts and graphics.	45.0
Diabetic Recipes: Great Recipes for Diabetics	Diet.	Figures, texts, and audio.	45.0
DiabetesDocs	Physical activity, diet, BP ^†^ , blood glucose and insulin.	Texts, graphics, and notepad.	40.0
*Diabetes Ginástica*	Physical activity.	Texts, images, and videos explaining the exercises.	37.5
Sukar – Blood Glucose & Diabetes Tracker App ^||^	Water intake, blood glucose, carbohydrate count, HbA1c ^‡^ , weight and medications.	Texts, pictures, and report sharing.	25.0

^*^ SUS = System Usability Scale;

^†^ BP = Blood Pressure;

^‡^ HbA1c = Glycated Hemoglobin;

^¶^ BD = Becton Dickinson;

^**^ BG = Blood Glucose;

^††^ NFC = Near Field Communication

^§^ DM = Diabetes Mellitus;

^||^ App = Application;

## Discussion

The interpretation of the broader data indicates a predominance of apps aimed at managing blood glucose and drug therapy, and a categorization of acceptable usability, followed by borderline. From this, it can be inferred that there is a need to invest in improving these apps to ensure that the user can make better use of the resource, broadening its support possibilities. In addition, the predominance of the vision focused on blood glucose and pharmacological control may emphasize the biological and professional-centered vision, minimizing the alternatives of a more active role for the person, especially related to behaviors and beliefs related to broader concepts of health.

 However, the content and resources found in the “apps for blood glucose management and drug therapy”, “apps for lifestyle changes”, and “apps for well-being and education measures” have, in a complementary way, direct implications for attitudes to self-care. This is because self-care attitudes are determined by behavioral, cognitive, and emotional issues ^(^ ^16^ ^-^ ^17^ ^)^ , and these contents and resources facilitate the development of positive health behaviors by including reminders/alarms, monitoring diaries, reports, and graphs. They use cognitive attitudes based on access to guidance and health goals, which reinforce positive emotional responses through motivational advice and rewarding good results. Thus, the person with DM can be guided to use more than one app from the different axes, in order to exploit the complementary features. 

 The features of the apps classified under the heading “Apps for managing blood glucose and drug therapy” include support for managing variations in blood glucose and the use of medication. Some studies have investigated the impact of these features on coping with the challenges of achieving individual glycemic targets, on decision-making regarding blood glucose patterns, and on the use of medication. A systematic review that evaluated the effect of smartphone apps on glycemic control in young patients with Type 1 DM (DM1) did not reveal a significant reduction in glycated hemoglobin (HbA1c) compared to usual care in combination. However, auxiliary apps with insulin or carbohydrate calculators were found to be beneficial in reducing HbA1c ^(^ ^18^ ^)^ . 

 In parallel, a real-world investigation indicated that the continuous use of blood glucose management apps is associated with the achievement of glycemic goals in clinical practice ^(^ ^19^ ^)^ . In participants with DM2, users who logged into an app for 19 days and had at least 85 days from their first to their last login had a more than twofold increase in the chance of reducing their absolute HbA1c value by 0.5% ^(^ ^20^ ^)^ . Part of these contributions may be associated with the different functions found in the apps, which is why it is important to consider the functionality of the apps so that there are better possibilities for patients to use them. It follows from this process that the quality of the dynamics in the use of health apps is significantly influenced by the functionality of these technologies ^(^ ^21^ ^)^ . 

In terms of content, some apps have gone beyond the usual glycemic monitoring, with the use of reminders, diaries, statistics, graphs, and user reports, making it possible to make appropriate adjustments and more accurate decision-making, given the scope of the information obtained, and thus improve the functionality of the apps to enhance their efficacy and effectiveness.

 With regard to resources that help with drug treatment, the tools that seek to help with drug adherence stand out. The contributions of these resources to treatment adherence are not yet clear from the literature. An evaluation of high-quality apps available free to the public showed that almost half of them did not promote high or moderate medication adherence. However, the limitation imposed by the small sample size and the lack of standardization of assessment tools on medication adherence is noteworthy ^(^ ^22^ ^)^ . 

 The “Apps for lifestyle changes” axis included apps with content and resources aimed at practicing physical activities, eating, blood pressure, and weight control. The identification of apps that cover this axis is in line with an investigation into the use of mobile devices by patients living with DM, which showed that a significant proportion of participants use apps for nutritional and dietary planning (85.5%), monitoring glycemic control (76.5%) and scheduling health appointments (90.5%) ^(^ ^23^ ^)^ . 

 In addition to this evidence, a systematic review of the effectiveness of lifestyle-related mobile apps for people with DM found that managing lifestyle habits through apps can help achieve effective glycemic control in the short term. However, there were no significant contributions to blood pressure or weight control ^(^ ^7^ ^)^ . 

 The axis “Apps for well-being and education measures” covered tools that helped set goals, educational guidelines, motivational advice, and encouragement when living with the chronicity of DM. Motivation is an important factor in people with DM being able to cope effectively with the disease ^(^ ^24^ ^)^ . Notably, these aspects are observed in the apps analyzed, including the educational theme, which is also relevant to maintaining the health of people with DM. In this context, apps seem to emerge as a tool capable of providing educational guidance and relevant information to individuals instantly, contributing to their teaching-learning process and the management of their health condition ^(^ ^8^ ^)^ . 

 Although motivational aspects were the aim of some of the apps, no mental health content was identified. It is known that people with DM are more susceptible to depression, anxiety, social isolation, and eating disorders ^(^ ^25^ ^)^ . In addition to the impact that treatment causes, such as physical and emotional stress on the person and their family, living with this chronic condition is permeated by numerous challenges, including adapting to a new care routine ^(^ ^4^ ^,^ ^25^ ^-^ ^26^ ^)^ . 

 It is also noteworthy that other important topics for the health of people with DM were not addressed by the apps analyzed, such as sexuality and reproduction. Sexuality is an essential component for maintaining a healthy life, and biological, psychological, and social repercussions can be influenced by DM ^(^ ^27^ ^)^ . Other topics that were little explored were educational guidelines on the complications of DM, such as diabetic neuropathy and peripheral arterial disease. Information on these complications and the prevention of skin lesions is fundamental for health management, helping to reduce health costs and avoiding hospital admissions for preventable causes, which can result in a better quality of life ^(^ ^28^ ^)^ . 

 Another important aspect is the lack of apps aimed at people with disabilities. No content was identified relating to visual impairment or low visual acuity resulting from another common complication of DM, such as diabetic retinopathy. The lack of apps on the subject is alarming, given that the contributions of technological advances to the inclusion of people with disabilities in society have already been recognized, minimizing inequalities, breaking down barriers, encouraging autonomy, and self-care, and ensuring greater accessibility ^(^ ^29^ ^)^ . This recognizes the need for investment in the development of tools for these issues. 

 Despite the above, it should be noted that among the plurality of 23 countries developing the selected apps, only two are Ibero-American (Brazil and Spain), whose total number of apps did not exceed 11 (2%). This reality may indicate an important indicator of technological investment related to the health of people with DM, above all because it corresponds to realities with different socio-cultural characteristics, the effects of which may suggest different impacts on more vulnerable populations in which functional, cultural and technological literacy and literacy have different realities between peoples and nations ^(^ ^30^ ^-^ ^31^ ^)^ , which is no different for people with DM. 

Furthermore, of the 71 apps selected, only 16 have a Portuguese version and one in Spanish. Of these apps, none were characterized as having excellent usability; the majority were considered to have acceptable usability (58.8%). This highlights the need for more and better discussions in Ibero-America on technologies for the health of people with DM, with a view to bringing information on blood glucose management, drug therapy, lifestyle changes, and well-being measures closer to the plural and objective reality of this population.

From a technical perspective, it can be seen that the versions of the apps analyzed were growing, indicating that the content was being updated and improved, with the addition of functionalities. It was also observed that the number of downloads was very varied, which is equivalent to the number of people who access the virtual store and download; however, this number may not reflect the actual number of app users, as merely downloading them does not automatically make the individual a user of the app’s content and resources.

 It is worth noting that although the vast majority of apps were in English, with 53/71, the number of downloads compared to Portuguese-language apps, with only 16/71, was not as great as the difference between these two modalities. For the English-language apps, the total number of downloads was >2,217,500, while the Portuguese-language apps had >1,960,100 downloads. This reality may reveal different behaviors among social groups from different cultures, regarding the importance they attach to health practices and/or the use of technologies for different purposes, which includes accessing information and establishing health-related routines. In this sense, Brazil, for example, is the country whose users spend the most time using their smartphone (Android) each day, with 5.4 hours/day, as revealed by the AppAnnie platform report of 2021 ^(^ ^32^ ^)^ . 

 This study found that most of the apps had acceptable usability or the features had some flaws, but with usability standards that were still acceptable. These results corroborate the data found in a systematic review that identified satisfaction rates for specialists and patients ranging from 38% to 80% ^(^ ^12^ ^)^ . The main usability problems are multi-step tasks, limited functionality and interaction, and difficult system navigation. Another study evaluating free mobile apps in Spanish identified few free DM management apps available, most of which lacked quality certification and few of which provided scientific references on their content ^(^ ^13^ ^)^ . 

This research provides a comprehensive overview of mobile apps aimed at the health of people with DM available on the main platforms, as well as an analysis of their features, content, and usability. This overview identified the limitations of the resources and the issues that still require development. In view of the results, nursing and health professionals and researchers can extract elements that facilitate both the detailed evaluation of apps and the safer recommendation of those that would best meet certain criteria, as well as directing investment in the development of new technologies and mobile resources necessary for the health management of people with DM.

A limitation of this study is that it did not include paid apps, which required specific resources or authorization from their developers. As the study focused on usability aspects, there is a limit to the reliability of the content used by developers and compliance with quality certification criteria, which requires future studies and an assessment of these parameters by health professionals who recommend apps.

## Conclusion

The mobile apps for the health of people with DM available on the main operating systems have varied content and resources. Most of them have acceptable usability and mainly involve the topics of blood glucose management, diet, drug therapy, and insulin therapy. They have the potential to help and guide users in their daily management of the disease, as well as being a possible care strategy to be used by health professionals.

However, some topics were not well covered, such a mental, sexual, and reproductive health, which can contribute to the therapeutic plan from a holistic and comprehensive perspective. There was also a need to develop more inclusive apps, with content and resources aimed at people with DM who live with some kind of disability. Finally, the recommendation for use by professionals should be preceded by verification of the reliability of the information contained in the apps, an issue which, due to the purpose of this research, was not assessed.
