# Supplementary material for: Benchmarking mobile applications for the health of people with Diabetes Mellitus
Source: Rev Lat Am Enfermagem. 2024 Jul 5;32:e4221. doi: 10.1590/1518-8345.7182.4221 (PMC11251684; doi:10.1590/1518-8345.7182.4221)
Supplement: Translation

# Translation: *Benchmarking* de aplicativos móviles dirigidos a la salud de personas con Diabetes Mellitus ^*^

**DOI:** 10.1590/1518-8345.7182.4220

**Authors:** Rafael Oliveira Pitta Lopes, Sara Rodrigues Chagas, Eduardo da Silva Gomes, Joice Cesar de Aguiar Barbosa, Ítalo Rodolfo Silva, Marcos Antônio Gomes Brandão

**Affiliations:** ^1^ Universidade Federal do Rio de Janeiro, Escola de Enfermagem Anna Nery, Rio de Janeiro, RJ, Brasil.; ^2^ Universidade Federal do Rio de Janeiro, Instituto de Enfermagem do Centro Multidisciplinar, Macaé, RJ, Brasil.; ^3^ São Lucas Hospital de Clínicas, Macaé, Rio de Janeiro, RJ, Brasil.; ^4^ Becario de la Coordenação de Aperfeiçoamento de Pessoal de Nível Superior (CAPES), Brasil.; ^5^ Becario del Conselho Nacional de Desenvolvimento Científico e Tecnológico (CNPq), Brasil.

**Keywords:** Diabetes Mellitus, Aplicaciones Móviles, eHealth Strategies, Teleenfermería, Promoción de la Salud, Inclusión Digital

## Abstract

**Objetivo:**

mapear los contenidos y los recursos de los aplicativos móviles sobre el manejo de la Diabetes Mellitus y su usabilidad en los principales sistemas operacionales.

**Método:**

investigaciones de *benchmarking* . El mapeo de los *apps* , de los contenidos y recursos en las plataformas *Play Store* y *App Store* fue realizado a partir de una adaptación en la estructura de revisiones del objetivo del *Joanna Briggs Institute.* Para el análisis de la usabilidad, los aplicativos fueron comprobados por dos semanas; para esto, se utilizó el instrumento *System Usability Scale* , siendo que puntajes entre 50-67 puntos, son considerados limítrofes; entre 68-84, tienen usabilidad aceptable; y, arriba de 85, tienen excelente aceptación por el usuario y para realizar análisis estadística descriptiva.

**Resultados:**

los contenidos más prevalentes fueron el manejo de la glucemia capilar, alimentación, terapia medicamentosa oral e insulinoterapia. En cuanto a los recursos se obtuvo mayor frecuencia de diarios y gráficos. En relación a la usabilidad, 2 *apps* fueron considerados de excelente aceptación; 34, productos con usabilidad aceptable; 29, los *apps* pueden presentar algunas fallas, sin embargo tiene estándares de usabilidad todavía aceptables; y, 6 presentaron fallas sin condiciones de usabilidad.

**Conclusión:**

los contenidos y los recursos de aplicativos móviles abordan los puntos fundamentales para el manejo de la diabetes mellitus, con recursos de fácil utilización, usabilidad aceptable por los usuarios y con potencial para auxiliar en el manejo de la diabetes mellitus, en el día a día de los pacientes.

## Destacados

**(1)** Se evaluó sistemáticamente la usabilidad de aplicativos de plataformas digitales.

**(2)** La mayoría de los aplicativos para personas con Diabetes Mellitus tiene buena usabilidad.

**(3)** El estudio ofrece un panorama de los recursos disponibles en los aplicativos.

**(4)** Los aplicativos poseen potencial para auxiliar el manejo de la Diabetes Mellitus.

**(5)** Los aplicativos deben ser evaluados por criterios de confiabilidad para poder ser recomendados.

## Introducción

 La Diabetes Mellitus (DM) puede llevar a complicaciones graves en la salud de todos los grupos etarios, inclusive grupos jóvenes, siendo esas complicaciones los principales motivos del empeoramiento de la calidad de vida, incapacidad y muerte de personas con DM ^(^ ^1^ ^-^ ^2^ ^)^ . 

 El manejo de la salud de personas con DM debe priorizar el establecimiento de un estilo de vida saludable con hábitos y prácticas que favorezcan el bienestar y el enfrentamiento de los desafíos de lo cotidiano ^(^ ^3^ ^)^ . Este manejo requiere múltiples responsabilidades de la persona en el autoadministración de su cotidiano: consciencia sobre autopercepción; desarrollo de autonomía; administración de relaciones sociales y papeles en el ambiente de trabajo; y, gestión de relaciones afectivas, respuestas emocionales y apoyos. Así como administrar otras que exigen fuerte acompañamiento profesional como las relacionadas con la seguridad y adhesión al tratamiento medicamentoso y las variaciones de la glucemia y todas las eventuales respuestas biológicas o medicamentosas ^(^ ^3^ ^)^ . 

 En la literatura existen informaciones de que el uso de las tecnologías para la DM mejora los resultados de salud, con reducción de barreras para informarse sobre a las orientaciones en salud, principalmente por intermedio de los dispositivos móviles; los que permiten el libre acceso y centralizan funciones, en apenas un dispositivo, lo que puede favorecer la autonomía y facilitar la administración de la enfermedad. Además, esas tecnologías pueden contribuir, positivamente, en la toma de decisiones sobre el monitoreo y manejo de la glucemia, automatizando procesos cotidianos o mejorando la solución de problemas relacionados a la salud ^(^ ^4^ ^-^ ^7^ ^)^ . 

 A pesar de que existen ventajas en el uso de los dispositivos móviles, también existen evidencias de barreras para el uso de aplicativos ( *apps* ) por personas con DM. Entre eses barreras pueden estar en la poca usabilidad, la ausencia de recursos de accesibilidad y en los contenidos presentes en los *apps* , formando obstáculos para los usuarios ^(^ ^8^ ^)^ . En adición, tanto en la práctica clínica profesional como en lo cotidiano de los usuarios, la identificación de estos *apps* no ocurre de forma sistematizada. La selección de estos recursos debe ser sustentada por un análisis eficiente del contenido, recursos y usabilidad, garantizando el uso seguro de estos instrumentos para el manejo de la salud. De ese modo, estudios de evaluación de desempeño de tecnologías son utilizados para analizar y evaluar las tecnologías y así, suministrar elementos para una interpretación de beneficios y limitaciones de la tecnología ^(^ ^9^ ^-^ ^11^ ^)^ . 

 Una revisión sistemática, publicada en 2017, sobre usabilidad y eficacia clínica de aplicativos móviles, para adultos con Diabetes Mellitus Tipo 2 (DM2), mostró que veinte publicaciones sobre la usabilidad, satisfacción del usuario y problemas de uso, indicaron una limitada efectividad, de aplicativos móviles, en promover el control glucémico ^(^ ^12^ ^)^ . En 2019, un estudio presentó resultados de la identificación de *apps* disponibles en español, en las plataformas *Play Store* ( *Android* ) y *App Store-iPhone Operating System* (iOS), indicando treinta y cuatro *apps* , cuya minoría (3) no disponía de certificación de calidad y pocos (3) indicaban las referencias científicas utilizadas ^(^ ^13^ ^)^ . 

Delante de lo expuesto, los desafíos permanecen en la identificación de aplicativos móviles disponibles para el manejo de la DM, así como en la verificación de sus propiedades de usabilidad. De ese modo, este artículo tiene el objetivo de mapear los contenidos y los recursos de los aplicativos móviles, sobre el manejo de la Diabetes Mellitus y su usabilidad en los principales sistemas operacionales.

## Método

### Delineamiento del estudio

 Se trata de un estudio de *benchmarking* ^(^ ^10^ ^-^ ^11^ ^)^ de aplicativos móviles, que buscó, por medio de un mapeo en plataformas de aplicativos, hacer un levantamiento de los contenidos, recursos y usabilidad de los aplicativos móviles, dirigidos a la salud de personas con DM. 

 El estudio de *benchmarking* permite evaluar el desempeño y resultados de un recurso o tecnología, realizar comparaciones con otros recursos, con el objetivo de proporcionar mejorías e ideas innovadoras; este estudio actúa como un instrumento de búsqueda que permite el perfeccionamiento e idealizaciones modernas de los *apps* con mejores evidencias y funcionalidades ^(^ ^10^ ^-^ ^11^ ^)^ . 

 El *scoping review framework* , según el *Joanna Briggs Institute* , fue adaptado para mapear los *apps* y sintetizar sus contenidos y recursos. Las etapas metodológicas adaptadas fueron: 1) Definición y alineamiento del objetivo y de la pregunta; 2) Desarrollo y alineamiento de los criterios de inclusión con el objetivo y la pregunta; 3) Descripción del abordaje planificado para búsqueda, selección, extracción de los contenidos y recursos, y presentación de los *apps* ; 4) Búsqueda de los *apps* ; 5) Selección de los *apps* ; 6) Extracción de los contenidos y recursos; 7) Análisis de los contenidos, recursos y usabilidad; 8) Presentación de los resultados; y, 9) Resumen en relación al propósito, conclusiones e implicaciones de las conclusiones. 

 El objetivo fue identificar: los *apps* móviles, dirigidos a la salud de personas con DM, sus contenidos, recursos y usabilidad. Para esto, se utilizó la pregunta: “¿Cuáles son los contenidos, recursos y usabilidad de los *apps,* dirigidos a la salud de personas con DM?”. Para alcanzar el objetivo los criterios de inclusión fueron los *apps* en los idiomas portugués, inglés y español, que incluyesen la temática de salud y la DM. Fueron excluidos aquellos que: 1) Los que exigían pagar para instalarlos; 2) Los destinados a profesionales de la salud; 3) Los que necesitaban la liberación de los autores para ser usados (número de registro y seña); 4) Los destinados al acompañamiento de evento/conferencia científica; 5) Los que eran juegos y evaluadores de conocimiento; 6) Los destinados a la salud de animales; 7) Los indisponibles para *download* o uso; 8) Los que necesitaban sensores específicos para su funcionamiento. El abordaje de búsqueda fue orientado por los términos “diabetes mellitus” y “salud”. 

 La búsqueda, selección y *download* fueron realizados en el período entre noviembre y diciembre de 2021, en las tiendas virtuales *Play Store* y *App Store* , de los sistemas operacionales *Android* ( *Google* ) e iOS ( *Apple* ), respectivamente. Dos investigadores realizaron de forma independiente la búsqueda y *download* de los *apps* en las tiendas virtuales. El primer investigador utilizó el *Samsung Galaxy* A71 compatible con *Android* y un *iPhone* 6s, compatible con iOS. El segundo investigador utilizó un *Xiaomi* 8 *Lite* compatible con *Android* y un *iPhone* 6s compatible con iOS. Cuatro búsquedas fueron realizadas en cada tienda virtual, empleando individualmente cada uno de los términos previamente establecidos. Después de la investigación, los *apps* fueron seleccionados por título y descripción resumida. Los *apps* que estuvieron de acuerdo con los criterios de inclusión y exclusión, fueron bajados y analizados. 

 Para la recolección y organización de las informaciones extraídas, cada investigador organizó los datos de los *apps* en una matriz de informaciones en el *Microsoft Excel* ^®^ , con descripción de: identificación (nombre, desarrollador, autor(es) responsable(es), año de lanzamiento, idioma, país de origen); público-objetivo (paciente, familiares/cuidadores y estudiantes); y, datos del *app* (descripción, versión actual, número de *downloads* , clasificación/evaluación). 

 En la séptima etapa, dos investigadores realizaron el análisis de la usabilidad de cada *app,* utilizando pruebas que durante dos semanas. Este período de evaluación fue necesario para que algunos *apps* pudiesen producir un conjunto, mínimo de datos, capaz de generar gráficos para el usuario. Al final de ese período, los investigadores disponían de elementos suficientes para analizar con seguridad la usabilidad. 

### Análisis de los datos de usabilidad

 El análisis de la usabilidad de los *apps* fue basado en el cuestionario *System Usability Scale* (SUS), el que fue validado para ser utilizado en Brasil en 2010 ^(^ ^14^ ^)^ . El SUS evalúa productos, *softwares* , aplicaciones, interfaces y *websites,* usando criterios de eficiencia, efectividad y satisfacción, que permiten identificar las ventajas y fragilidades, por medio del uso previo de los *apps* seleccionados. El cuestionario posee diez ítems que muestran una visión integral del usuario en relación al sistema; el usuario debe seleccionar un ítem de una escala que va de 1 (Discuerdo Completamente) a 5 (Concuerdo Completamente) ^(^ ^14^ ^)^ . 

 Para el cálculo de la usabilidad, fue realizada la substracción de 1 de la puntuación para las respuestas impares y de 5 para las respuestas pares. Para obtener la media final se multiplicó el valor encontrado por 2,5, lo que produjo la puntuación final (entre 0 y 100). En relación a los puntajes del SUS, la puntuación entre 50-67 puntos es considerada limítrofe, es decir, el recurso puede presentar algunas fallas; sin embargo, posee estándares de usabilidad todavía aceptables. Las puntuaciones entre 68-84 representan productos con usabilidad aceptable. Por otro lado, los *softwares* que puntúan arriba de 85 poseen excelente aceptación por el usuario ^(^ ^15^ ^)^ . Después de la evaluación independiente, los dos investigadores compararon los resultados de la aplicación del SUS para cada *app* , siendo debatidas las posibles divergencias de puntuaciones, en los diez ítems del cuestionario; ese debate fue mediado por la revisión concomitante del *app* por los dos investigadores hasta obtención de consenso para la determinación de la puntuación final de usabilidad del *app* . 

### Aspectos éticos

 Este estudio no fue sometido a la apreciación del Comité de Ética en Investigación, por caracterizarse como investigación en que no participan directamente seres humanos, en que los datos son de dominio público; también, no contenía ningún conflicto de interés comercial, profesional o de cualquier otra modalidad. Las informaciones recolectadas en los *apps* fueron utilizadas exclusivamente para una finalidad científica. Los *apps* analizados fueron debidamente mencionados en este estudio. 

## Resultados

 Fueron identificados de 431 *apps* en las plataformas de aplicativos, siendo 269 en la *Play Store* y 162 en la *App Store* . De estos, 30 fueron excluidos por estar duplicados, restando 401 *apps* . Después del análisis de los títulos y descripción de los contenidos, 255 *apps* fueron seleccionados para *download* y evaluación minuciosa. Finalmente, la muestra fue constituida por 71 *apps* . El proceso de selección de los *apps* está descrito en la Figura 1 . 

 Los *apps* analizados quedaron disponibles en las plataformas a partir del año de 2010, con aumento en el número de desarrollos a partir de 2013. Se observó que 2020 fue el período con mayor número de lanzamientos, totalizando 12 (16,9%) *apps* . Entre tanto, se identificó disminución en el desarrollo de *apps* después del año de 2021. 

 La mayoría de los *apps* tenía desarrolladores de los Estados Unidos (21; 29,5%) y de la India (14; 19,7%). A continuación, se identificaron *apps* de Brasil (6; 8,4%); Francia (4; 5,6%); Alemania (3; 4,2%); Paquistán, España, Rusia, Canadá y China (2; 2,8%) cada uno; Austria, Grecia, Polonia, Suiza, Turquía, Reino Unido, Países Bajos, Bulgaria, Dinamarca, Japón, Italia, Finlandia y Bélgica (1; 1,4%) cada uno. 

 El manejo de la diabetes fue abordado en todos los *apps* , que incluían contenidos como: autoadministración; control y gerenciamiento de los estándares glucémicos; registro de las actividades físicas; orientaciones educativas relacionadas a la alimentación; informaciones acerca del uso de medicaciones e insulina; y, control de la presión y medidas antropométricas ( Figura 2 ). 

**Figura 1:** - Diagrama de flujo de la selección de los aplicativos en las plataformas virtuales seleccionadas. Macaé, RJ, 2023

**Figura 2:** - Caracterización de los aplicativos según el idioma, objetivos, versión, evaluación y *downloads* . Macaé, RJ, 2023

**Aplicativo**	**Idioma**	**Descripción/** **Objetivo**	**Versión**	**Estrellas/** **evaluación**	**Downloads**
*mySugr*	Portugués	Administración de la DM ^*^ a través de registros diarios	3.92.10	4,5	>1.000.000
*Diabetes: Management & Blood Sugar Tracker App* ^†^	Portugués	Acompañar, analizar y mantener la DM ^*^ bajo control	8.0.11	4,4	>500.000
*Blood Sugar Diary*	Inglés	Diario de la glucemia	1.1.1	4,0	>500.000
*Blood Glucose Tracker*	Inglés	Registrar niveles de glucemia	1.8.12	4,7	>500.000
*Diabete – Diário Glucose*	Inglés	Diario de la glucemia	4.4	4,6	>500.000
*A Minha Glycemia*	Portugués	Herramienta para monitoreo de la glucosa	1.8.2	4,2	>100.000
BD ^‡^ *Diabetes Care*	Portugués	Administración de la DM ^*^	3.1.2	3,3	>100.000
*Beat Diabetes*	Inglés	Recetas y consejos de alimentación	9.9	4,7	>100.000
*Blood Sugar Log – Diabetes Tracker*	Inglés	Rastrear los registros de glucemia, PA ^§^ , peso y medicamentos	1.13	4,5	>100.000
Control de la Glucemia	Español	Diario de la glucemia	2.0.3	4,1	>100.000
*Diabéticos Receitas*	Portugués	Herramienta con recetas saludables	45.0.0	4,1	>100.000
*Diabetes Diary – Blood Glucose Tracker*	Inglés	Rastrear niveles de glucosa, PA ^§^ , peso y HbA1c ^||^	1.26	4,7	>100.000
*Diário Diabetes*	Inglés	Diario glucémico y de la HbA1c ^||^	2.2.9	3,9	>100.000
*Intellin Diabetes Log Tracker & Risk Manager App* ^†^	Inglés	Administración de la DM ^*^	1.10.167	4,0	>100.000
*Social Diabetes*	Portugués	Controlar mejor a través de registros de datos	4.17.53	4,0	>100.000
BG ^¶^ *Monitor Diabetes*	Inglés	Administración del uso de la insulina y la práctica de ejercicios físicos	8.0.1	4,3	>50.000
*Diabetes Controle*	Portugués	Diario de insulina	1.5	4,6	>50.000
*Diabetes Tracker* *Gratuito*	Inglés	Diario de la glucemia	1.9	3,9	>50.000
*Glycemic Index*	Inglés	Índice glucémico alimenticio	2.15.17	4,5	>50.000
*Blood Glucose Tracker*	Inglés	Administración de la DM ^*^	2.0	4,3	>10.000
*Diabetes Control App* ^†^	Inglés	Almacenar y administrar datos para control de la DM ^*^	3.4.1.0	4,1	>10.000
Diabetes Ginástica	Inglés	Rutinas de ejercicios para usuarios diabéticos	1.3	3,9	>10.000
*Diabetes Logbook – Blood Glucose Tracker*	Inglés	Mantener diario de la glucemia y contaje de carbohidratos	2.7	3,8	>10.000
*Diabetic Recipes: Great Recipes for Diabetics*	Inglés	Recetas para diabéticos	2.0	4,7	>10.000
*Diário de sangue*	Portugués	Diario de la glucemia	2.2.0	3,9	>10.000
*forDiabetes* : *Aplicativo de Diabetes*	Diversos	Registrar y monitoreo de datos esenciales para control de la DM ^*^	1.119.4	4,5	>10.000
*Glucômetro – Rastreamento de Diabetes*	Inglés	Rastrear los niveles de glucosa, HbA1c ^||^ e insulina administrada	2.16	4,2	>10.000
*Glucool Diabetes*	Inglés	Administración de la DM ^*^	1.4.3.1	3,7	>10.000
*My Sugar Diary – Diabetes App* ^†^	Inglés	Registrar los niveles glucémicos	1.8.6	4,2	>10.000
*Blood Glucose Tracker – Track Your Blood Glucose*	Inglés	Administración de la DM ^*^ a través del registro de niveles de glucosa, peso y PA ^§^	1.9.2	4,2	>5.000
*Diabetes Food Recipes*	Inglés	Recetas para diabéticos	3.1.4	4,2	>1.000
*Sugar – Blood Glucose & Diabetes Tracker App* ^†^	Inglés	Administración de la DM ^*^	1.1.6	3,7	>1.000
*Diabetes Test My Blood Sugar Diary – BP* ^**^ *Glucose*	Inglés	Administración de niveles de glucosa, HbA1c ^||^ , peso y PA ^§^ en forma de estadísticas	04.04.2021	4,8	>1.000
*Wecheck – Diabetes Lifelog*	Inglés	Administración de la DM ^*^	1.0.9	4,1	>1.000
*Blue Circle Diabetes*	Inglés	Administración de la DM ^*^	1.25	4,8	>1.000
*Diabetes Tracker App: Blood Glucose & Cholesterol*	Inglés	Registrar y rastrear los niveles de glucosa y colesterol	1.0	3,9	>1.000
*Diabetes Tracker*	Inglés	Administración de la DM ^*^	1.0	4,0	>1.000
*Joddi – Diabetes LogBook*	Inglés	Diario para control de la glucemia	0.7.2	4,1	>1.000
*Eglucomonitor – Sugar & Diabetes Monitor App* ^†^	Inglés	Monitoreo diario de la glucemia	1.0.1	NI ^††^	>500
*Blood Sugar Diary - Blood Glucose Tracker*	Inglés	Diario de la glucemia	1.2	4,8	>100
*Butterfly – Diabetes App* ^†^	Inglés	Herramienta con metas saludables semanales para control de la DM ^*^	10.0	NI ^††^	>100
*Diabetic Diet Plan*	Inglés	Recetas para diabéticos	1.3	NI ^††^	>100
*Diabetes Meal Plan*	Inglés	Plan de alimentaciones para individuos con DM ^*^	1.0	NI ^††^	>100
*Receitas para diabéticos*	Portugués	Alimentos y recetas para diabéticos	1.00	4,9	>100
*Diabetic Diet Plan*	Inglés	Plan alimenticio para diabéticos	1.0	NI ^††^	>50
*Smart Diabetes Diary*	Inglés	Registrar valores de la glucemia, insulina y medicamentos	1.0	NI ^††^	>10
*Carbok: Carb Counting & Management for Diabetics*	Inglés	Realizar contaje de carbohidratos	2.1.0	NI ^††^	>5
*Blood Sugar Diary for Diabetes*	Inglés	Diario de la glucemia	2.11	NI ^††^	NI ^§^
*Center Health – The Diabetes App* ^†^	Inglés	Diario de la glucemia	3.4	NI ^††^	NI ^§^
*Contagem de Carboidratos*	Portugués	Realizar contaje de carbohidratos	2.1	NI ^††^	NI ^††^
*DiabetesDocs*	Inglés	Herramienta para realizar el manejo de la DM ^*^	4.6	NI ^††^	NI ^††^
*Diabética Comida Receitas* *App* ^†^	Portugués	Recetas saludables para diabéticos	4.1	NI ^††^	NI ^††^
*Diabetes Diary*	Inglés	Herramienta para control de la glucemia, insulina y carbohidratos ingeridos	2.1	NI ^††^	NI ^††^
*Diabetes Health Manager*	Inglés	Administración de la DM ^*^	10.7	NI ^††^	NI ^††^
*Diabetes Pilot Pro*	Inglés	Diario de la glucemia	7.0	NI ^††^	NI ^††^
*Diabetes Pro*	Inglés	Administración de la DM ^*^	1.5	NI ^††^	NI ^††^
*Diabetes SmartManager*	Inglés	Herramienta para cálculo de dosis de insulina	1.1	NI ^††^	NI ^††^
*Dits*	Inglés	Herramienta de diario alimenticio y de glucemia, con realización de contaje de carbohidratos	1.4	NI ^††^	NI ^††^
*Glic Diabetes e Glucemia*	Portugués	Diario de la glucemia	4.1	NI ^††^	NI ^††^
*GlicAPP*	Portugués	Herramienta para recordatorio de registro y medición de la glucemia	1.3	5,0	NI ^††^
*Glicose Companheiro*	Inglés	Diario de la glucemia	7.2	NI ^††^	NI ^††^
*glucoSecrets*	Inglés	Diario de la glucemia	5.2	NI ^††^	NI ^††^
*GlucoseMonitor*	Inglés	Herramienta para control de la glucemia, peso y medicamentos	3.10	4,5	NI ^††^
*gluQUO: Control Your Diabetes*	Inglés	Herramienta para administrar la DM ^*^	2.3	NI ^††^	NI ^††^
*Happy Bob*	Inglés	Herramienta con consejos alentadores para el control de la DM ^*^	1.9	NI ^††^	NI ^††^
*Health2Sync*	Inglés	Diario de la glucemia	2.6	NI ^††^	NI ^††^
*Help Diabetes*	Inglés	Herramienta para realizar contaje de carbohidratos	3.1	NI ^††^	NI ^††^
*iGlicho*	Portugués	Administración de la DM ^*^	0.8	NI ^††^	NI ^††^
*Insulin Calculator*	Portugués	Realizar cálculo de dosis de insulina	1.0	NI ^††^	NI ^††^
*Os pacientes diabéticos, acompanhar e monitorar seus níveis de glicose no sangue*	Portugués	Administración de la DM ^*^	1.6	NI ^††^	NI ^††^
*Undermyfork: Diabetes*	Inglés	Diario de alimentación para individuos con DM ^*^	2.12	NI ^††^	NI ^††^

^‡^ BD = *Becton Dickinson* ;

^¶^ BG = *Blood Glucose* ;

^**^ BP = *Blood Pressure* ;

^||^ HbA1c = Hemoglobina Glicosilada a;

^§^ PA = Presión Arterial;

^†^ *App* = Aplicativo;

^*^ DM = Diabetes Mellitus;

^††^ NI = No Identificado

Tres ejes temáticos pueden representar los contenidos y recursos que indican el propósito central de los apps: “aplicativos para gestión de la glucemia y de la terapia medicamentosa”, engloban acciones de gerenciamiento de la fisiopatología y tratamiento de la DM; “aplicativos para el cambio en el estilo de vida” como: actividades físicas, alimentación, control de la presión y del peso; y, “aplicativos para medidas de bienestar y educación”, conteniendo: establecimiento de metas, orientaciones motivacionales, estímulo frente a la convivencia con la cronicidad de la DM y orientaciones educativas.

 La Figura 3 presenta la categorización de los *apps* analizados en lo que se refiere a los ejes temáticos y a los temas abordados. Los temas predominantes fueron manejo (55; 77,4%), seguido de alimentación (41; 57,7%), terapia medicamentosa oral e insulinoterapia (39; 54,9%). Algunos aplicativos contemplan más de un tema en su contenido, y permiten que se clasifiquen en una o más categorías. 

**Figura 3:** - Categorización de los *apps* en cuanto a los ejes temáticos y temas abordados. Macaé, RJ, 2023

 En relación a los recursos disponibles en los *apps* , fueron identificados: diarios (50; 70,4%); gráficos (46; 64,7%); figuras/imágenes (37; 52,1%); informes (27; 38%); recordatorios/alarmas (17; 23,9%); estadísticas (7; 9,8%); y, tablas (5; 7%). También fueron identificados recursos relacionados a los hábitos de vida y bienestar: registro de la presión arterial (22; 30,9%), peso (14; 19,7%), monitoreo de actividades físicas (15; 21,1%) y orientaciones/metas que auxilian a la gestión eficaz de la DM (2; 2,8%). Algunos *apps* permitían la inserción de datos sobre el perfil sociodemográfico del usuario (47; 66,2%) y clínico (52; 73,2%), como el año del diagnóstico (9;12,6%) y el tipo de DM (24; 33,8%). 

 En lo que se refiere a la usabilidad de los *apps* analizados, dos (2,8%) obtuvieron un puntaje ≥ 85; 34 (47,9%) puntuaron entre 68-84 puntos; 29 (40,8%) presentaron puntajes entre 50-67 puntos; seis (8,5%) obtuvieron puntajes inferiores a 50. La Figura 4 ilustra las informaciones de los *apps* sobre el contenido, los recursos y la usabilidad. 

**Figura 4:** - Caracterización de los aplicativos seleccionados en cuanto a su contenido, recursos y usabilidad. Macaé, RJ, 2023

**Aplicativo**	**Contenido**	**Recursos**	**Usabilidad** **(puntos en el SUS** ^*^ **)**
Usabilidad excelente (n=2)			
*forDiabetes* : *Aplicativo de diabetes*	Glucemia, medicaciones, PA ^†^ , alimentaciones, actividad física, peso, HbA1c ^‡^ y artículos sobre la DM ^§^ .	Textos, figuras, gráficos, recordatorios, intercambio de informes, sincronización con otros dispositivos; inserción de actividades diarias, alimentaciones, medicaciones, niveles de glucemia, PA ^†^ , HbA1c ^‡^ y peso.	85,0
*Carbok: Carb Couting & Management for Diabetic*	Alimentación e conteo de carbohidratos.	Figuras, textos, calculadora, gráficos; permite registrar las características de las alimentaciones.	85,0
Usabilidad aceptable (n=37)			
*mySugr*	Diario de la glucemia, administración de medicamentos, insulina e ingestión de carbohidratos.	Textos, gráficos e figuras. Permite adicionar recordatorios, registrar glucemia, pasos/actividad física, insulina/medicaciones, peso y enviar informes.	82,5
*Diabetes: Management & Blood Sugar Tracker App* ^||^	Registro de la glucemia, administración de medicamentos, alimentos, peso, PA ^†^ , actividad física y exámenes de laboratorio.	Textos, gráficos y figuras. Posibilita registros de glucemia, alimentos y medicamentos, definición de alarmas, además de acompañar la evolución por medio de gráficos y de informes.	82,5
*A minha glycemia*	Glucemia capilar, insulina, HbA1c ^‡^ y PA ^†^ .	Inserir valores de glucemia, recordatorios, tablas, histogramas, bloque de notas y adicionar contactos.	82,5
*Blood Glucose Tracker*	Alimentación, peso, PA ^†^ , glucemia y medicamentos.	Textos, gráficos, figuras y bloques de notas.	82,5
*Center Health – The Diabetes App* ^||^	Diario de la glucemia, insulina, conteo de carbohidratos, ejercicios y sueño.	Textos, gráficos, figuras, registro de datos sincronización con sensores, importación y exportación de planillas.	82,5
*Contagem de Carboidratos*	Conteo de carbohidratos.	Gráficos, textos y calculadora.	82,5
*Glic Diabetes e Glucemia*	Glucemia, insulina, PA ^†^ , medicamentos y artículos educacionales.	Figuras, textos, gráficos, alerta de hipoglucemia, tabla nutricional, cálculo de insulina, conteo de carbohidratos, conecta la ficha electrónica y envía el informe de los datos.	82,5
*iGlicho*	Alimentación, diario de glucemia, conteo de carbohidratos e insulina.	Gráficos, textos, tabla de alimentos, conteo de carbohidratos, cálculo de insulina y generación de informes.	82,5
*Controle de Glicose*	Glucemia, HbA1c ^‡^ , insulina, medicamentos, PA ^†^ y alimentación.	Gráficos, textos. Posibilita adicionar exámenes de laboratorio, visualizar los niveles de glucosa y PA ^†^ en gráficos y exportar los informes.	80,0
*Smart Diabetes Diary*	Glucemia, insulina y medicamentos.	Figuras, gráficos, estadísticas, textos, bloque de notas y exportación de datos.	80,0
*Diabetes Logbook – Blood Glucose Tracker*	Diario de la glucemia, HbA1c ^‡^ , conteo de carbohidratos y insulina.	Textos, gráficos y figuras.	80,0
*Diabética Comida Receitas App* ^||^	Alimentación.	Textos e imágenes.	80,0
*Health2Sync*	Diario de la glucemia, alimentación, conteo de carbohidratos, actividad física, PA ^†^ y peso.	Gráficos, textos, figuras. Permite adicionar fotos de las alimentaciones y generar informes.	80,0
*Dits*	Glucemia capilar y artículos educacionales.	Gráficos, textos, figuras y artículos.	80,0
*Blood Sugar Log – Diabetes Tracker*	PA ^†^ , glucemia, Hb1Ac ^‡^ , peso y medicamentos.	Figuras, textos y envio de informes.	77,5
*Diabetes Meal Plan*	Alimentación.	Imágenes, textos y calculadora.	77,5
*Blood Glucose Tracker*	Glucemia, PA ^†^ , medicamentos, Hb1Ac ^‡^ y peso.	Gráficos, estadísticas, textos y bloque de notas.	77,5
*Diabetes Tracker App* ^||^ : *Blood Glucose & Cholesterol*	PA ^†^ , peso, glucemia, HbA1c ^‡^ y colesterol.	Textos, recordatorios y gráficos.	77,5
*Diabetes Tracker*	Actividad física, alimentación, medicamentos, PA ^†^ y glucemia.	Figuras, recordatorios, investigación por voz, adicionar lista de profesionales, fecha de consultas y de informes.	77,5
*Glicose Companheiro*	Glucemia y peso.	Gráficos, estadísticas, textos, calculadora e informes.	77,5
*Happy Bob*	Autovigilancia glucémica, metas y consejos para control de la DM ^§^ .	Textos, figuras.	77,5
*Blood Sugar Diary for Diabetes*	Autovigilancia glucémica.	Textos, estadísticas, notificaciones, gráficos, alteración del tema, artículos y sincronización con los sensores.	77,5
*SocialDiabetes*	Señales vitales, diario de la glucemia, HbA1c ^‡^ , medicamentos, alimentación, actividad física, estímulo y contenidos educacionales.	Textos, calculadora, estimativa de la HbA1c ^‡^ , alarmas, sincronización con los sensores y exportación de informes.	75,0
*Diabetes Health Manager*	Síntomas, medicamentos, tratamiento y monitoreo glucémico.	Figuras, textos, gráficos y artículos.	75,0
*Diabéticos Receitas*	Recetas saludables.	Textos y figuras. Permite favorecer recetas, crear listas de compras y buscar recetas por medio de la selección de un ingrediente/alimento específico.	72,5
*Blue Circle Diabetes*	Actividad física, alimentación, insulina y glucemia.	Textos, imágenes, calculadora, tienda virtual, informaciones nutricionales, recetas y encuentros *on-line* sobre la DM ^§^ .	72,5
*Eglucomonitor – Sugar & Diabetes Monitor App* ^||^	Diario de la glucemia e insulina.	Textos y gráficos.	72,5
*Diabetes Diary*	Glucemia, conteo de carbohidratos e insulina.	Gráficos, textos y calculadora.	72,5
*BD* ^¶^ *Diabetes Care*	Registro de la glucemia e insulina; recetas saludables, preguntas y respuestas sobre la DM ^§^ y los contenidos educacionales.	Textos y figuras. Posibilita el registro de glucemia, insulina, intercambio de informes, recordatorios, conexión con los otros dispositivos, preguntas y respuestas, investigar recetas y contenidos educacionales y definir metas diarias.	70,0
*Diabetes Diary – Blood Glucose Tracker*	PA ^†^ , peso, HbA1c ^‡^ , medicamentos y glucemia.	Textos, gráficos e informes.	70,0
*Intellin Diabetes Log Tracker & Risk Manager App*	Actividad física, PA ^†^ , glucemia e insulina.	Textos, figuras, gráficos, sincronización con los otros dispositivos y intercambio de informes.	70,0
*Glycemic Index*	Alimentación.	Textos y herramientas para adicionar a los alimentos.	70,0
*Undermyfork: Diabetes*	Alimentación y diario glucémico.	Textos, imágenes y gráficos.	70,0
*Insulin Calculator*	Insulina.	Textos y gráficos. Permite crear alimentaciones, calcular insulina y obtener histórico.	70,0
*BG* ^**^ *Monitor Diabetes*	Diario de la glucemia, insulina, actividad física y alimentación.	Gráficos y textos. Posee recordatorios e informes.	67,5
*Diabetes Tracker* *Gratuito*	Diario glucémico.	Textos, gráficos y recordatorios.	67,5
*Receitas para Diabéticos*	Alimentación.	Imágenes y textos.	67,5
Usabilidad limítrofe (n=26)			
*Diabetes Control App* ^||^	Alimentación, insulina, medicamentos y glucosa sanguínea.	Textos y gráficos. Recordatorio, bloque de notas, adicionar contacto de profesionales, *chat* e informes.	65,0
*Diabetes Controle*	Autovigilancia glicémica.	Gráficos, estadísticas, figuras, textos e intercambio de informes.	65,0
*Blood Glucose Tracker – Track Your Blood Glucose*	Diario glucémico, PA ^†^ y peso.	Textos, gráficos y exportación de datos.	65,0
*Glucômetro – Rastreamento de Diabetes*	Diario de la glucemia, insulina y HbA1c ^‡^ .	Inserir los niveles de glucosa, HbA1c ^‡^ e insulina.	65,0
*Diabetes Test My Blood Sugar Diary – BP* ^††^ *Glucose*	Autovigilancia glucémica, PA ^†^ , peso, HbA1c ^‡^ y medicaciones.	Figuras, textos, gráficos y exportación de informes.	65,0
*Blood Sugar Diary - Blood Glucose Tracker*	Alimentación, medicamentos y glucemia.	Textos, figuras, gráficos y artículos sobre la DM ^§^ .	65,0
*GlucoseMonitor*	Diario glucémico y señales vitales.	Gráficos, textos y recordatorios.	65,0
*gluQUO: Control Your Diabetes*	Alimentación, actividad física y diario de la glucemia.	Textos, figuras, gráficos, recordatorios, calculadora; permite adicionar fotos de las alimentaciones y obtener informes para intercambiar.	65,0
*GlicAPP*	Exámenes de laboratorio, glucemia e insulina.	Textos. Posibilita registrar datos médicos, exámenes de laboratorio y sugestiones.	65,0
*My Sugar Diary – Diabetes App* ^||^	Diario glucémico.	Textos, tablas y figuras.	62,5
*Diabetes Pro*	Diario de la glucemia y medicamentos.	Figuras, textos, gráficos y herramienta para realizar rotación de datos para verificación de la glucemia capilar.	62,5
*Diabetes Food Recipes*	Alimentación.	Textos e imágenes.	60,0
*Diabetes Pilot Pro*	Diario glucémico, alimentación y conteo de carbohidratos.	Textos y gráficos.	60,0
*Os pacientes diabéticos, acompanhar e monitorar seus níveis de glicose no sangue*	Diario de la glucemia.	Textos, tablas, gráficos y recordatorios.	60,0
Diario Diabetes	Actividad física, alimentación, PA ^†^ , peso, glucosa, HbA1c ^‡^ y medicamentos.	Textos, gráficos, medias de los datos inseridos y recordatorios.	57,5
*Glucool Diabetes*	Glucemia, insulina, PA ^†^ , conteo de carbohidratos, HbA1c ^‡^ , ejercicios y medicaciones.	Gráficos, textos, calculadora y exportación de datos.	57,5
*Joddi – Diabetes LogBook*	Diario de la glucemia e insulina.	Textos, registrar y exportar datos.	57,5
*Diabetic Diet Plan* (v. 1.0)	Alimentación.	Textos e imágenes.	57,5
*Diabetic Diet Plan* (v. 1.3)	Alimentación.	Textos e imágenes.	57,5
*glucoSecrets*	Diario de la glucemia, actividad física y medicamentos.	Gráficos/estadísticas, textos y figuras.	57,5
*Help Diabetes*	Conteo de carbohidratos.	Textos y calculadora.	57,5
*Beat Diabetes*	Alimentación.	Imágenes y textos.	52,5
Diabete – Diario Glucose	Glucemia y peso.	Gráficos, estadísticas, recordatorios, inserir valores, exportar e importar datos.	52,5
*Wecheck – Diabetes Lifelog*	Señales vitales, alimentación, insulina y glucemia.	Gráficos, textos, registro de señales vitales, glucosa e insulina; conexión NFC ^‡‡^ , *Bluetooth* y sincronización con aparatos *Wecheck* .	52,5
*Butterfly – Diabetes App* ^||^	Metas semanales para control de la DM ^§^ .	Textos y registro de datos.	52,5
*Diabetes SmartManager*	Conteo de carbohidratos, insulina y diario de la glucemia.	Textos, recordatorios, gráficos. Permite registrar la glucemia e insulina, modificar el color del tema y obtener informes.	50,0
Usabilidad inadecuada (n=6)			
*Di* á *rio de Sangue*	Diario glucémico, PA ^†^ y peso.	Gráficos, calendario y registro de glucemia, PA ^†^ y peso.	47,5
*Blood Sugar Diary*	Diario de la glucemia.	Textos y gráficos.	45,0
*Diabetic Recipes: Great Recipes for Diabetics*	Alimentación.	Figuras, textos y audios.	45,0
*DiabetesDocs*	Actividad física, alimentación, PA ^†^ , glucemia e insulina.	Textos, gráficos y bloque de notas.	40,0
*Diabetes Ginástica*	Actividad física.	Textos, imágenes y videos explicativos de los ejercicios.	37,5
*Sukar – Blood Glucose & Diabetes Tracker App* ^||^	Ingestión hídrica, glucemia, conteo de carbohidratos, HbA1c ^‡^ , peso y medicaciones.	Textos, figuras e intercambio de informes.	25,0

^*^ SUS = *System Usability Scale* ;

^¶^ BD = *Becton Dickinson* ;

^**^ BG = *Blood Glucose* ;

^††^ BP = *Blood Presure* ;

^‡‡^ NFC = *Near Field Communication.*

^§^ DM = *Diabetes Mellitus* ;

^†^ PA = Presión Arterial;

^||^ *App* = Aplicativo;

^‡^ HbA1c = Hemoglobina glucosilada;

## Discusión

La interpretación más amplia de los datos indica la predominancia de aplicativos orientados para la gestión de la glucemia, de la terapia medicamentosa y de una categorización de usabilidad aceptable, seguida de la limítrofe. Así, podemos inferir que existe la necesidad de realizar inversiones en el perfeccionamiento de estos aplicativos para garantizar que el usuario pueda utilizar mejor el recurso, ampliando sus posibilidades de soporte. Además, la predominancia de la visión enfocada en la glucemia y en el control farmacológico, puede enfatizar la visión de la biología y centrada en los profesionales, minimizando las alternativas del papel más activo del usuario, especialmente relacionadas a los comportamientos y creencias habituales de conceptos más ampliados de la salud.

 Entretanto, los contenidos y los recursos encontrados en los “aplicativos para la gestión de la glucemia y de la terapia medicamentosa”, “aplicativos para los cambios en el estilo de vida”, y “aplicativos para las medidas de bienestar y educación” poseen, de forma complementaria, implicaciones directas en las actitudes para el autocuidado. Esto se debe a que las actitudes de autocuidado son determinadas por cuestiones comportamentales, cognitivas y emocionales ^(^ ^16^ ^-^ ^17^ ^)^ , y estos contenidos y recursos facilitan el desarrollo de comportamientos de salud positivos, por incluir recordatorios/alarmas, diarios de monitoreo, informes y gráficos. Los *Apps* utilizan actitudes cognitivas basadas en el acceso a orientaciones y metas de salud, que refuerzan las respuestas emocionales positivas, por medio de consejos motivacionales y recompensas por los buenos resultados alcanzados. Así, la persona con DM puede ser orientada a utilizar más de un aplicativo de los diferentes ejes, de forma a explorar las características complementarias. 

 En relación a los recursos de los *apps* clasificados en el eje “Aplicativos para la gestión de la glucemia y de la terapia medicamentosa”, estos englobaron desde el apoyo al manejo de las variaciones de la glucemia, hasta la utilización de los medicamentos. Algunas investigaciones se enfocaron en el impacto de estos recursos en el enfrentamiento a los desafíos para alcanzar las metas glucémicas individuales, para la toma de decisiones frente a los estándares de la glucemia y para el uso de las medicaciones. Una revisión sistemática que evaluó el efecto de los *apps* para *smartphones,* en el control glucémico en pacientes jóvenes con DM Tipo 1 (DM1), no reveló una reducción significativa de la Hemoglobina Glicosilada (HbA1c), cuando comparada a los cuidados habituales combinados. Sin embargo, se identificó que *apps* auxiliares con las calculadoras de insulina o carbohidratos fueron benéficos en la reducción de la HbA1c ^(^ ^18^ ^)^ . 

 En paralelo, una investigación del tipo mundo real indicó que, el uso continuo de *apps* de administración de la glucemia está asociado al alcance de metas glucémicas, en la práctica clínica ^(^ ^19^ ^)^ . Por otro lado, entre los participantes con DM2, los usuarios que realizaron *login* en un *app* por 19 días y tuvieron, por lo menos, 85 días desde el primero hasta el último *login,* tuvieron aumento de más de dos veces la probabilidad de reducir el valor absoluto de HbA1c en 0,5% ^(^ ^20^ ^)^ . Parte de estas contribuciones puede estar asociada a las diferentes funciones encontradas en los *apps* , razón por la cual es importante considerar la funcionalidad de los *apps,* para que existan mejores posibilidades de utilización por parte de los pacientes. De este proceso se desprende el entendimiento de que, la calidad de la dinámica en el uso de *apps* para la salud sufre la influencia significativa de la funcionalidad de esas tecnologías ^(^ ^21^ ^)^ . 

 En relación a los contenidos, algunos *apps* fueron más allá del monitoreo glucémico usual, con la introducción de recordatorios, diarios, estadísticas, gráficos e informes del usuario; así, sería posible hacer ajustes adecuados y toma de decisiones más cuidadosas, frente a la amplitud de las informaciones obtenidas y, así, mejorar la funcionalidad de los *apps* para potencializar su eficacia y efectividad. 

 En lo que se refiere a los recursos que auxilian el tratamiento medicamentoso, se destacan las herramientas que buscan auxiliar en la adhesión medicamentosa. Todavía no están evidentes, en la literatura, las contribuciones de estos recursos para la adhesión al tratamiento. La evaluación de *apps,* de alta calidad disponibles gratuitamente para el público, demostró que casi la mitad de estos aplicativos no promovieron la alta o moderada adhesión medicamentosa. Sin embargo, se destaca la limitación impuesta por la muestra reducida y la falta de estandarización de los instrumentos evaluadores, sobre la adhesión medicamentosa ^(^ ^22^ ^)^ . 

 El eje “Aplicativos para el cambio en el estilo de vida” contempló *apps* con contenidos y recursos dirigidos a la práctica de actividades físicas, alimentación, control de la presión y del peso. La identificación de *apps* que contemplan este eje se coaduna con una investigación sobre el uso de dispositivos móviles por pacientes que conviven con DM; esta evidenció que una parcela significativa de los participantes utiliza *apps* para la planificación nutricional y dietética (85,5%), monitoreo del control glucémico (76,5%) y programación de consultas de salud (90,5%) ^(^ ^23^ ^)^ . 

 Adicionado a estas evidencias, una revisión sistemática - sobre la efectividad de *apps* móviles relacionados al estilo de vida de personas con DM - detectó que la administración de hábitos de vida por medio de *apps* puede auxiliar a alcanzar, en el corto plazo, el control glucémico efectivo. Sin embargo, no se observaron contribuciones significativas en el control de la presión arterial y del peso ^(^ ^7^ ^)^ . 

 El eje “Aplicativos para las medidas de bienestar y educación” abarcó herramientas que auxilian el estabelecimiento de metas, orientaciones educativas, consejos motivacionales e incentivadores, frente a la convivencia con la cronicidad de la DM. Se destaca la capacidad de la motivación que es muy importante para que las personas con DM consigan lidiar de manera eficaz con la enfermedad ^(^ ^24^ ^)^ . Es relevante destacar que esos aspectos son observados en los *apps* analizados, incluyendo la temática educacional, que también se caracteriza como importante para la manutención de la salud de personas con DM. En ese contexto, los *apps* parecen surgir como una herramienta capaz de suministrar orientaciones educativas e informaciones relevantes para los individuos de forma instantánea, contribuyendo con el proceso de enseñanza-aprendizaje y el manejo de su cuadro de salud ^(^ ^8^ ^)^ . 

 A pesar de que los aspectos motivacionales fueron el objetivo de algunos *apps* , no se identificaron contenidos dirigidos a la salud mental. Se sabe que personas con DM son más susceptibles a la depresión, a la ansiedad, al aislamiento social y a los trastornos alimenticios ^(^ ^25^ ^)^ . Además del impacto que el tratamiento causa, como estrés físico y emocional en la persona y en la familia, la convivencia con esa condición crónica es impregnada por numerosos desafíos, que incluyen la adaptación a una nueva rutina de cuidados ^(^ ^4^ ^,^ ^25^ ^-^ ^26^ ^)^ . 

 También, se señala que otras temáticas importantes, para la salud de personas con DM, no fueron abordadas por los *apps* analizados, como la sexualidad y reproducción. La sexualidad es un componente esencial para la manutención de una vida saludable y la DM ^(^ ^27^ ^)^ puede ocasionar repercusiones biológicas, psicológicas y sociales. Otras temáticas poco exploradas fueron las orientaciones educativas sobre las complicaciones de la DM, como la neuropatía diabética y la Enfermedad Arterial Periférica. Las informaciones relativas a esas complicaciones y a la prevención de lesiones cutáneas, son fundamentales para el manejo de la salud y contribuyen para la reducción de los costos en salud, evitando internaciones hospitalarias por causas prevenibles; así, esas informaciones son importantes para obtener una mejor calidad de vida ^(^ ^28^ ^)^ . 

 Otro aspecto importante es la falta de aplicativos dirigidos a personas con deficiencia. No fueron identificados contenidos relacionados a la deficiencia visual o baja acuidad visual, que son provenientes de otra complicación común de la DM, como la retinopatía diabética. La ausencia de aplicativos sobre este tema es alarmante, una vez que son reconocidas las contribuciones de los avances tecnológicos para la inclusión de la persona con deficiencia en la sociedad, minimizando las desigualdades, eliminando barreras, estimulando la autonomía, el autocuidado y garantizando una mayor accesibilidad ^(^ ^29^ ^)^ . Así, se reconoce la necesidad de realizar inversiones en el desarrollo de herramientas para esas temáticas. 

 A pesar de lo expuesto, cabe destacar que, en medio a la pluralidad de 23 países desenvolvedores de los *apps* seleccionados, apenas dos son iberoamericanos (Brasil y España), cuya expresión total del número de *apps* no ultrapasó 11 (2%). Esa realidad puede señalar un importante indicador de la necesidad de realizar inversiones tecnológicas, relacionadas a la salud de la persona con DM, sobre todo, por corresponder a las realidades con características socioculturales distintas, cuyas consecuencias pueden provocar impactos distintos en poblaciones más vulnerables, en las cuales la alfabetización funcional, cultural, tecnológica y el analfabetismo imprimen realidades diferentes entre los pueblos y naciones ^(^ ^30^ ^-^ ^31^ ^)^ , lo que no es diferente para las personas con DM. 

 Además, del total de 71 *apps* seleccionados, apenas 16 presentaron versión en portugués y uno en español. Entre esos *apps* , ninguno fue caracterizado con excelente aceptación de usabilidad, siendo la mayoría considerada de usabilidad aceptable (58,8%). Frente a esto, se coloca en evidencia la necesidad de tener más y mejores discusiones en el ámbito iberoamericano sobre las tecnologías para la salud de personas con DM, con el objetivo de aproximar las informaciones sobre el manejo de la glucemia, la terapia medicamentosa, los cambios de estilo de vida y las medidas de bienestar de la realidad plural y objetiva de esa población. 

 Dentro de una perspectiva técnica, se constata que el número de las versiones de los *apps* analizados era creciente, indicando la actualización y el perfeccionamiento de los contenidos, apuntando adición de funcionalidades. Además, se observó que el número de *downloads* era muy variado, lo que equivale al número de personas que accede a la tienda virtual y realiza el *download* ; sin embargo, ese número puede no reflejar la cantidad real de usuarios de los *apps* , ya que apenas bajarlos no torna al individuo, automáticamente, usuario de los contenidos y recursos del aplicativo. 

 Cabe destacar que, a pesar de la expresiva mayoría de los *apps* presentó la versión en inglés, con 53/71, el número de *downloads* cuando comparado con los *apps* de lengua portuguesa, de apenas 16/71, no fue tan superior en cuanto a la diferencia entre esas dos modalidades. Para los *apps* en lengua inglesa, el número total de *downloads* fue > 2.217.500, en cuanto que los *apps* en portugués sumaron >1.960.100 *downloads* . Esa realidad puede revelar comportamientos diferentes entre grupos sociales de culturas distintas, sobre la importancia que atribuyen a las prácticas de salud y/o de la utilización de tecnologías para diferentes finalidades, lo que incluye acceder a las informaciones y establecer las rutinas relacionadas a la salud. En ese sentido, Brasil, por ejemplo, es el país cuyos usuarios demandan más tiempo de uso diario de *smartphone* ( *Android* ), con 5,4 horas/día, conforme reveló el informe de la plataforma *AppAnnie* , de 2021 ^(^ ^32^ ^)^ . 

 En este estudio se verificó que la mayoría de los *apps* presentaba usabilidad aceptable y los recursos presentaban algunas fallas, pero con estándares de usabilidad todavía aceptables. Estos resultados son corroborados con los datos encontrados en una revisión sistemática que identificó los índices de satisfacción de especialistas y pacientes entre 38% y 80% ^(^ ^12^ ^)^ . Los principales problemas de usabilidad son tareas de varias etapas, de funcionalidad, de interacciones limitadas y de navegación difícil en el sistema. Otro estudio que evaluó los aplicativos móviles en español, identificó pocos aplicativos gratuitos disponibles para el control de la DM, siendo la mayoría sin certificación de calidad y pocos informaban referencias científicas sobre su contenido ^(^ ^13^ ^)^ . 

 Esta investigación presenta un panorama amplio sobre los *apps* móviles, dirigidos a la salud de personas con DM, disponibles en las principales plataformas, así como un análisis sobre sus recursos, contenidos y usabilidad. Este panorama identificó las limitaciones de los recursos y los temas que todavía requieren desarrollo. Con estos resultados, los profesionales e investigadores de la Enfermería y del área de la salud, pueden extraer elementos que facilitan tanto la evaluación detallada de los *apps,* como la recomendación más segura de aquellos que mejor atenderían determinados criterios; también permite orientar las inversiones en el desarrollo de nuevas tecnologías y recursos móviles, necesarios para el manejo de la salud de personas con DM. 

 Como limitación de este estudio, se menciona la no inclusión de los *apps* pagados, que exigían recursos específicos o autorización de sus desarrolladores. Como el estudio se centró en los aspectos de la usabilidad, existe la limitación de la confiabilidad del contenido empleado por los desarrolladores y la observancia de los criterios de certificación de calidad; estas limitaciones exigen realizar nuevos estudios y la evaluación de estos parámetros, para que los profesionales de la salud puedan recomendar los *apps* . 

## Conclusión

 Los *apps* móviles dirigidos a la salud de personas con DM disponibles, en los principales sistemas operacionales, poseen contenidos y recursos variados; estos presentan, en su mayoría, una usabilidad aceptable y contienen principalmente las temáticas del manejo de la glucemia, alimentación, terapia medicamentosa e insulinoterapia. Además, poseen potencial para auxiliar y orientar a los usuarios en el manejo de la enfermedad cotidianamente, también, se constituyen en una posible estrategia de cuidado a ser utilizada por los profesionales de salud. 

 Sin embargo, algunas temáticas fueron poco contempladas, como la salud mental, sexual y reproductiva, las que podrían contribuir para el plan terapéutico, dentro de una perspectiva holística e integral. También, se evidenció la necesidad de desarrollar *apps* más inclusivos, con contenidos y recursos dirigidos a personas con DM que conviven con algún tipo de deficiencia. Por último, la recomendación de uso por los profesionales debe ser antecedida por la verificación de la confiabilidad de las informaciones contenidas en los *apps* , aspecto que, debido a la finalidad de esta investigación, no fue evaluada. 

# Translation: *Benchmarking* de aplicativos móveis voltados à saúde de pessoas com *Diabetes Mellitus* ^*^

**DOI:** 10.1590/1518-8345.7182.4222

**Authors:** Rafael Oliveira Pitta Lopes, Sara Rodrigues Chagas, Eduardo da Silva Gomes, Joice Cesar de Aguiar Barbosa, Ítalo Rodolfo Silva, Marcos Antônio Gomes Brandão

**Affiliations:** ^1^ Universidade Federal do Rio de Janeiro, Escola de Enfermagem Anna Nery, Rio de Janeiro, RJ, Brasil.; ^2^ Universidade Federal do Rio de Janeiro, Instituto de Enfermagem do Centro Multidisciplinar, Macaé, RJ, Brasil.; ^3^ São Lucas Hospital de Clínicas, Macaé, Rio de Janeiro, RJ, Brasil.; ^4^ Bolsista da Coordenação de Aperfeiçoamento de Pessoal de Nível Superior (CAPES), Brasil.; ^5^ Bolsista do Conselho Nacional de Desenvolvimento Científico e Tecnológico (CNPq), Brasil.

**Keywords:** Diabetes Mellitus, Aplicativos Móveis, Estratégias de eSaúde, Telenfermagem, Promoção da Saúde, Inclusão Digital

## Abstract

**Objetivo:**

mapear os conteúdos e os recursos dos aplicativos móveis sobre o manejo da *Diabetes Mellitus* e sua usabilidade nos principais sistemas operacionais.

**Método:**

pesquisa de *benchmarking* . O mapeamento dos *apps* , dos conteúdos e dos recursos nas plataformas *Play Store* e *App Store* foi realizado a partir de uma adaptação na estrutura de revisões de escopo da *Joanna Briggs Institute.* Para a análise da usabilidade, os aplicativos foram testados por duas semanas e utilizou-se o instrumento *System Usability Scale* , sendo que escores entre 50-67 pontos, é considerado limítrofe, entre 68-84, produtos com usabilidade aceitável e acima de 85, excelente aceitação pelo usuário e, para a análise, estatística descritiva.

**Resultados:**

os conteúdos mais prevalentes foram o manejo da glicemia capilar, alimentação, terapia medicamentosa oral e insulinoterapia. Quanto aos recursos, obteve-se a maior frequência de diários e gráficos. Em relação à usabilidade, dois *apps* foram considerados de excelente aceitação; 34, produtos com usabilidade aceitável; 29, o recurso pode apresentar algumas falhas, mas tem padrões de usabilidade ainda aceitáveis e 6, com falhas sem condições de usabilidade.

**Conclusão:**

os conteúdos e os recursos de aplicativos móveis abordam os pontos fundamentais para o manejo da *Diabetes Mellitus* com recursos de fácil utilização, com usabilidade aceitável pelos usuários e tem potencial para auxiliar no manejo da *Diabetes Mellitus* no dia a dia dos pacientes.

## Destaques

**(1)** Avaliou-se sistematicamente a usabilidade de aplicativos de plataformas digitais.

**(2)** A maioria dos aplicativos para pessoas com *Diabetes Mellitus* tem boa usabilidade.

**(3)** O estudo oferece um panorama dos recursos disponíveis nos aplicativos.

**(4)** Os aplicativos possuem potencial para auxiliar no manejo da *Diabetes Mellitus.*

**(5)** Os aplicativos devem ser avaliados por critério de confiabilidade para recomendação.

## Introdução

 A *Diabetes Mellitus* (DM) pode levar a complicações graves na saúde de todos os grupos etários, inclusive grupos jovens, sendo essas complicações os principais motivos da piora da qualidade de vida, da incapacidade e morte de pessoas com DM ^(^ ^1^ ^-^ ^2^ ^)^ . 

 O manejo da saúde de pessoas com DM deve priorizar o estabelecimento de um estilo de vida saudável com hábitos e práticas que favorecem o bem-estar e o enfrentamento dos desafios do cotidiano ^(^ ^3^ ^)^ . Tal manejo requer múltiplas responsabilidades da pessoa no autogerenciamento de seu cotidiano, consciência sobre o autoconceito, desenvolvimento de autonomia, gerenciamento das relações sociais e dos papéis no ambiente de trabalho, das relações afetivas e de respostas emocionais, e de apoio; bem como outras que exigem forte acompanhamento profissional como aquelas relacionadas à segurança e à adesão ao tratamento medicamentoso, às variações da glicemia e todas as eventuais respostas biológicas ou medicamentosas ^(^ ^3^ ^)^ . 

 Na literatura existem indicações de que o uso das tecnologias para a DM melhora os desfechos de saúde com redução de barreiras no acesso às orientações em saúde, principalmente por intermédio dos dispositivos móveis; com livre acesso e centralização de funções em apenas um dispositivo que pode favorecer a autonomia e facilitar o gerenciamento da doença. Ademais, tais tecnologias podem contribuir positivamente na tomada de decisão sobre a monitorização e no manejo da glicemia, automatizando os processos cotidianos ou melhorando a solução de problemas relacionados à saúde ^(^ ^4^ ^-^ ^7^ ^)^ . 

 Embora existam vantagens no uso dos dispositivos móveis, também há evidências de barreiras para o uso de aplicativos ( *apps* ) entre as pessoas com DM. Entre essas barreiras podem estar a pouca usabilidade, a ausência de recursos de acessibilidade e os conteúdos presentes nos *apps* , constituindo-se, portanto, em entraves para os usuários ^(^ ^8^ ^)^ . Em adição, tanto na prática clínica profissional quanto no cotidiano dos usuários, a identificação destes *apps* não ocorre de forma sistematizada. A seleção destes recursos deve ser sustentada por uma análise eficiente do conteúdo, recursos e da usabilidade, garantindo o uso seguro destes instrumentos para o manejo da saúde. Desse modo, os estudos de avaliação de desempenho de tecnologias são utilizados para analisar e avaliar as tecnologias e, com isso, fornecer elementos para uma interpretação de benefícios e limitações da tecnologia ^(^ ^9^ ^-^ ^11^ ^)^ . 

 Uma revisão sistemática, publicada em 2017, sobre a usabilidade e a eficácia clínica de aplicativos móveis para os adultos com *Diabetes Mellitus* Tipo 2 (DM2) mostrou que vinte publicações sobre a usabilidade, satisfação do usuário e os problemas de uso, indicaram limitada efetividade de aplicativos móveis em promover o controle glicêmico ^(^ ^12^ ^)^ . Em 2019, um estudo apresentou resultados da identificação de *apps* disponíveis em espanhol nas plataformas *Play* *Store* ( *Android* ) e *App Store-iPhone Operating System* (iOS), indicando trinta e quatro *apps* , sendo que três não dispunham de certificação de qualidade e apenas três indicavam as referências científicas utilizadas ^(^ ^13^ ^)^ . 

Frente ao exposto, os desafios permanecem na identificação de aplicativos móveis disponíveis para o manejo da DM, bem como na verificação de suas propriedades de usabilidade. Desse modo, este artigo tem o objetivo mapear os conteúdos e os recursos dos aplicativos móveis sobre o manejo da DM e sua usabilidade nos principais sistemas operacionais.

## Método

### Delineamento do estudo

 Trata-se de um estudo de *benchmarking* ^(^ ^10^ ^-^ ^11^ ^)^ de aplicativos móveis, o qual buscou, por meio de um mapeamento em plataformas de aplicativos, fazer um levantamento dos conteúdos, dos recursos e da usabilidade dos aplicativos móveis voltados à saúde de pessoas com DM. 

 Um estudo de *benchmarking* permite avaliar o desempenho e os resultados de um recurso ou tecnologia, realizar comparações com outros recursos, com vistas a proporcionar melhorias e ideias inovadoras. Atua como um instrumento de busca que permite o aperfeiçoamento e as idealizações modernas dos *apps* com as melhores evidências e funcionalidades ^(^ ^10^ ^-^ ^11^ ^)^ . 

 A *scoping review framework* , segundo o *Joanna Briggs Institute* , foi adaptada para mapear os *apps* e sintetizar seus conteúdos e recursos. As etapas metodológicas adaptadas foram: 1) Definição e alinhamento do objetivo e da pergunta; 2) Desenvolvimento e alinhamento dos critérios de inclusão com o objetivo e a pergunta; 3) Descrição da abordagem planejada para a busca, seleção, extração dos conteúdos e recursos, e apresentação dos *apps* .; 4) Procura pelos *apps* ; 5) Seleção dos *apps* ; 6) Extração dos conteúdos e recursos; 7) Análise dos conteúdos, recursos e da usabilidade; 8) Apresentação dos resultados e, 9) Resumo em relação ao propósito, conclusões e implicações das conclusões. 

 O objetivo foi identificar os *apps* móveis voltados à saúde de pessoas com DM, seus conteúdos, recursos e usabilidade. Para tal, desenvolveu-se a pergunta: “Quais são os conteúdos, recursos e usabilidade dos *apps* voltados à saúde de pessoas com DM?”. Para o alcance do objetivo, os critérios de inclusão foram: os *apps* nos idiomas português, inglês e espanhol, que compreendessem a temática de saúde e DM e foram excluídos aqueles que: 1) exigissem pagamento para a instalação; 2) destinados aos profissionais de saúde; 3) que necessitassem liberação dos autores para seu uso (número de cadastro e senha); 4) relacionados ao acompanhamento de evento/conferência científica; 5) jogos e avaliadores de conhecimento; 6) referentes à saúde de animais; 7) estivessem indisponíveis para *download* ou uso; 8) necessitassem de sensores específicos para o seu funcionamento. A abordagem de busca foi orientada pelos termos “ *diabetes mellitus* ” e “saúde”. 

 A procura, seleção e *download* foram realizados no período de novembro a dezembro de 2021, nas lojas virtuais *Play Store* e *App Store* , dos sistemas operacionais *Android* ( *Google* ) e iOS ( *Apple* ), respectivamente. Dois pesquisadores realizaram de forma independente a busca e *download* dos *apps* nas lojas virtuais. O primeiro pesquisador utilizou o *Samsung Galaxy* A71 compatível com *Android* ; e um *iPhone* 6s, compatível com iOS. Já o segundo pesquisador utilizou um *Xiaomi* 8 *Lite* , compatível com *Android* ; e um *iPhone* 6s, compatível com iOS. Quatro buscas foram empreendidas em cada loja virtual, empregando individualmente cada um dos termos previamente estabelecidos. Após a pesquisa, os *apps* foram selecionados via título e descrição resumida. Os *apps* que estiveram de acordo com os critérios de inclusão e exclusão foram baixados e analisados. 

 Para a coleta e organização das informações extraídas, cada pesquisador organizou os dados dos *apps* em uma matriz de informações no *Microsoft Excel* ^®^ , com descrição de identificação (nome, desenvolvedor, autor(es) responsável(is), ano de lançamento, idioma, país de origem); público-alvo (paciente, familiares/cuidadores e estudantes); dados do *app* (descrição, versão atual, número de *downloads* , classificação/avaliação). 

 Em seguida, dois pesquisadores realizaram a análise da usabilidade de cada *app* em testes que duraram duas semanas. Este período de avaliação foi necessário para que alguns *apps* pudessem produzir um conjunto mínimo de dados capaz de gerar gráficos ao usuário. Ao final desse período, os pesquisadores dispunham de elementos suficientes para analisar a usabilidade com segurança. 

### Análise dos dados de usabilidade

 A análise da usabilidade dos *apps* foi baseada no questionário *System Usability Scale* do Sistema Único de Saúde (SUS), o qual foi validado para uso no Brasil em 2010 ^(^ ^14^ ^)^ . O SUS avalia produtos, *softwares* , aplicações, interfaces e *websites* por critérios de eficiência, efetividade e satisfação, permite a identificação das vantagens e das fragilidades por meio do uso prévio dos *apps* selecionados. O questionário contém dez itens que mostram uma visão integral do usuário em relação ao sistema, tendo o usuário que selecionar um item de uma escala que vai de 1 (Discordo Completamente) a 5 (Concordo Completamente) ^(^ ^14^ ^)^ . 

 Para o cálculo da usabilidade, foi realizada a subtração de 1 da pontuação para as respostas ímpares e para as respostas pares subtraiu-se o valor de 5. Para obter-se a média final, multiplicou-se o valor encontrado por 2.5, que produziu a pontuação final (entre 0 a 100). Em relação aos escores do SUS, a pontuação entre 50-67 pontos é considerada limítrofe, ou seja, o recurso pode apresentar algumas falhas, mas possui padrões de usabilidade ainda aceitáveis. As pontuações entre 68-84 representam produtos com usabilidade aceitável. Já os *softwares* que pontuam acima de 85 possuem excelente aceitação pelo usuário ^(^ ^15^ ^)^ . Após a avaliação independente, os dois pesquisadores compararam os resultados de aplicação do SUS para cada *app* , sendo debatidas as possíveis divergências de pontuações nos dez itens do questionário. Esse debate foi mediado pela revisão concomitante do *app* pelos dois pesquisadores até a obtenção de consenso para a determinação da pontuação final de usabilidade do *app* . 

### Aspectos éticos

 Este estudo não foi submetido à apreciação por Comitê de Ética em Pesquisa por caracterizar-se como pesquisa que não envolvesse diretamente seres humanos, cujos dados são de domínio público, e não envolveu quaisquer conflitos de interesse comerciais, profissionais ou de qualquer outra modalidade. As informações coletadas nos *apps* foram utilizadas exclusivamente para fins científicos. Os *apps* analisados foram devidamente mencionados no estudo. 

## Resultados

 Foram identificados 431 *apps* nas plataformas de aplicativos, sendo 269 na *Play Store* e 162 na *App Store* . Desses, 30 foram excluídos por estarem duplicados, restando 401 *apps* . Após a análise dos títulos e a descrição dos conteúdos, 255 *apps* foram selecionados para o *download* e avaliação minuciosa. Ao final, a amostra foi constituída por 71 *apps* . O processo de seleção dos *apps* está descrito na Figura 1 . 

 Os *apps* analisados foram disponibilizados nas plataformas a partir do ano de 2010, com ascensão no número de desenvolvimentos a partir de 2013. Observou-se que em 2020 foi o período com maior número de lançamentos, totalizando 12 (16,9%) *apps* . Entretanto, identificou-se um declínio no desenvolvimento de *apps* após o ano de 2021. 

 A maioria dos *apps* tinha desenvolvedores dos Estados Unidos (21; 29,5%) e da Índia (14; 19,7%). Na sequência, identificaram-se *apps* do Brasil (6; 8,4%); França (4; 5,6%); Alemanha (3; 4,2%); Paquistão, Espanha, Rússia, Canadá e China (2; 2,8% cada); Áustria, Grécia, Polônia, Suíça, Turquia, Reino Unido, Países Baixos, Bulgária, Dinamarca, Japão, Itália, Finlândia e Bélgica (1; 1,4%) cada um. 

**Figura 1:** - Fluxograma da seleção dos aplicativos nas plataformas virtuais selecionadas. Macaé, RJ, 2023

 O manejo da DM foi abordado em todos os *apps* , envolvendo conteúdos tais como: o automonitoramento, controle e gerenciamento dos padrões glicêmicos, registro das atividades físicas, orientações educativas relacionadas à alimentação, informações sobre o uso de medicações e insulina, bem como sobre o controle pressórico e as medidas antropométricas ( Figura 2 ). 

 Três eixos temáticos podem representar os conteúdos e os recursos que indicam o propósito central dos *apps* : “aplicativos para a gestão da glicemia e da terapia medicamentosa”, englobando as ações de gerenciamento da fisiopatologia e tratamento da DM; “aplicativos para a mudança no estilo de vida”, tais como: atividade física, alimentação, controle pressórico e de peso; e “aplicativos para as medidas de bem-estar e educação”, com o estabelecimento de metas, orientações motivacionais, encorajamento frente ao convívio com a cronicidade da DM e as orientações educativas. 

**Figura 2:** - Caracterização dos aplicativos segundo o idioma, objetivos, versão, avaliação e *downloads* . Macaé, RJ, 2023

**Aplicativo**	**Idioma**	**Descrição/** **Objetivo**	**Versão**	**Estrelas/** **avaliação**	**Downloads**
*mySugr*	Português	Gerenciar a DM ^*^ por meio de registros diários	3.92.10	4,5	>1.000.000
*Diabetes: Management & Blood Sugar Tracker App* ^†^	Português	Acompanhar, analisar e manter a DM ^*^ sob controle	8.0.11	4,4	>500.000
*Blood Sugar Diary*	Inglês	Diário da glicemia	1.1.1	4,0	>500.000
*Blood Glucose Tracker*	Inglês	Registrar os níveis da glicemia	1.8.12	4,7	>500.000
Diabete – Diário Glucose	Inglês	Diário da glicemia	4.4	4,6	>500.000
A Minha Glycemia	Português	Ferramenta para o monitoramento de glicose	1.8.2	4,2	>100.000
BD ^‡^ *Diabetes Care*	Português	Gerenciar a DM ^*^	3.1.2	3,3	>100.000
*Beat Diabetes*	Inglês	Receitas e dicas de alimentação	9.9	4,7	>100.000
*Blood Sugar Log – Diabetes Tracker*	Inglês	Rastrear os registros de glicemia, PA ^§^ , peso e medicamentos	1.13	4,5	>100.000
Controle de Glicose	Espanhol	Diário da glicemia	2.0.3	4,1	>100.000
Diabéticos Receitas	Português	Ferramenta com receitas saudáveis	45.0.0	4,1	>100.000
*Diabetes Diary – Blood Glucose Tracker*	Inglês	Rastrear os níveis de glicose, PA ^§^ , peso e HbA1c ^||^	1.26	4,7	>100.000
Diário Diabetes	Inglês	Diário glicêmico e da HbA1c ^||^	2.2.9	3,9	>100.000
*Intellin Diabetes Log Tracker & Risk Manager App* ^†^	Inglês	Gerenciar a DM ^*^	1.10.167	4,0	>100.000
Social Diabetes	Português	Controlar melhor por meio de registros de dados	4.17.53	4,0	>100.000
BG ^¶^ *Monitor Diabetes*	Inglês	Gerenciar o uso da insulina e a prática de exercícios físicos	8.0.1	4,3	>50.000
Diabetes Controle	Português	Diário de insulina	1.5	4,6	>50.000
*Diabetes Tracker* Gratuito	Inglês	Diário da glicemia	1.9	3,9	>50.000
*Glycemic Index*	Inglês	Índice glicêmico alimentar	2.15.17	4,5	>50.000
*Blood Glucose Tracker*	Inglês	Gerenciar a DM ^*^	2.0	4,3	>10.000
*Diabetes Control App* ^†^	Inglês	Armazenar e gerenciar os dados para o controle da DM ^*^	3.4.1.0	4,1	>10.000
Diabetes Ginástica	Inglês	Rotinas de exercícios para os usuários diabéticos	1.3	3,9	>10.000
*Diabetes Logbook – Blood Glucose Tracker*	Inglês	Realizar o diário da glicemia e a contagem de carboidratos	2.7	3,8	>10.000
*Diabetic Recipes: Great Recipes for Diabetics*	Inglês	Receitas para os diabéticos	2.0	4,7	>10.000
Diário de sangue	Português	Diário da glicemia	2.2.0	3,9	>10.000
*forDiabetes* : Aplicativo de Diabetes	Diversos	Registrar e monitorar os dados essenciais para o controle da DM ^*^	1.119.4	4,5	>10.000
Glucômetro – Rastreamento de Diabetes	Inglês	Rastrear os níveis de glicose, HbA1c ^||^ e insulina administrada	2.16	4,2	>10.000
*Glucool Diabetes*	Inglês	Gerenciar a DM ^*^	1.4.3.1	3,7	>10.000
*My Sugar Diary – Diabetes App* ^†^	Inglês	Registrar os níveis glicêmicos	1.8.6	4,2	>10.000
*Blood Glucose Tracker – Track Your Blood Glucose*	Inglês	Gerenciar a DM ^*^ por meio do registro de níveis de glicose, peso e PA ^§^	1.9.2	4,2	>5.000
*Diabetes Food Recipes*	Inglês	Receitas para os diabéticos	3.1.4	4,2	>1.000
*Sukar – Blood Glucose & Diabetes Tracker App* ^†^	Inglês	Gerenciar a DM ^*^	1.1.6	3,7	>1.000
*Diabetes Test My Blood Sugar Diary – BP* ^**^ *Glucose*	Inglês	Gerenciar os níveis de glicose, HbA1c ^||^ , peso e PA ^§^ em forma de estatísticas	04.04.2021	4,8	>1.000
*Wecheck – Diabetes Lifelog*	Inglês	Gerenciar a DM ^*^	1.0.9	4,1	>1.000
*Blue Circle Diabetes*	Inglês	Gerenciar a DM ^*^	1.25	4,8	>1.000
*Diabetes Tracker App: Blood Glucose & Cholesterol*	Inglês	Registrar e rastrear os níveis de glicose e colesterol	1.0	3,9	>1.000
*Diabetes Tracker*	Inglês	Gerenciar a DM ^*^	1.0	4,0	>1.000
*Joddi – Diabetes LogBook*	Inglês	Diário para o controle da glicemia	0.7.2	4,1	>1.000
*Eglucomonitor – Sugar & Diabetes Monitor App* ^†^	Inglês	Monitoramento diário da glicemia	1.0.1	NI ^††^	>500
*Blood Sugar Diary - Blood Glucose Tracker*	Inglês	Diário da glicemia	1.2	4,8	>100
*Butterfly – Diabetes App* ^†^	Inglês	Ferramenta com metas saudáveis semanais para o controle da DM ^*^	10.0	NI ^††^	>100
*Diabetic Diet Plan*	Inglês	Receitas para os diabéticos	1.3	NI ^††^	>100
*Diabetes Meal Plan*	Inglês	Plano de refeições para os indivíduos com DM ^*^	1.0	NI ^††^	>100
Receitas para diabéticos	Português	Alimentos e receitas para os diabéticos	1.00	4,9	>100
*Diabetic Diet Plan*	Inglês	Plano alimentar para os diabéticos	1.0	NI ^††^	>50
*Smart Diabetes Diary*	Inglês	Registrar os valores da glicemia, insulina e medicamentos	1.0	NI ^††^	>10
*Carbok: Carb Counting & Management for Diabetics*	Inglês	Realizar a contagem de carboidratos	2.1.0	NI ^††^	>5
*Blood Sugar Diary for Diabetes*	Inglês	Diário da glicemia	2.11	NI ^††^	NI ^§^
*Center Health – The Diabetes App* ^†^	Inglês	Diário da glicemia	3.4	NI ^††^	NI ^§^
Contagem de Carboidratos	Português	Realizar a contagem de carboidratos	2.1	NI ^††^	NI ^††^
*DiabetesDocs*	Inglês	Ferramenta para realizar o manejo da DM ^*^	4.6	NI ^††^	NI ^††^
Diabética Comida Receitas *App* ^†^	Português	Receitas saudáveis para os diabéticos	4.1	NI ^††^	NI ^††^
*Diabetes Diary*	Inglês	Ferramenta para realizar o controle da glicemia, insulina e carboidratos ingeridos	2.1	NI ^††^	NI ^††^
*Diabetes Health Manager*	Inglês	Gerenciar a DM ^*^	10.7	NI ^††^	NI ^††^
*Diabetes Pilot Pro*	Inglês	Diário da glicemia	7.0	NI ^††^	NI ^††^
*Diabetes Pro*	Inglês	Gerenciar a DM ^*^	1.5	NI ^††^	NI ^††^
*Diabetes SmartManager*	Inglês	Ferramenta para o cálculo de doses de insulina	1.1	NI ^††^	NI ^††^
*Dits*	Inglês	Ferramenta de diário alimentar e da glicemia, com a realização de contagem de carboidratos	1.4	NI ^††^	NI ^††^
Glic Diabetes e Glicemia	Português	Diário da glicemia	4.1	NI ^††^	NI ^††^
GlicAPP	Português	Ferramenta para lembrete de registro e medição da glicemia	1.3	5,0	NI ^††^
Glicose Companheiro	Inglês	Diário da glicemia	7.2	NI ^††^	NI ^††^
*glucoSecrets*	Inglês	Diário da glicemia	5.2	NI ^††^	NI ^††^
*GlucoseMonitor*	Inglês	Ferramenta para o controle da glicemia, peso e medicamentos	3.10	4,5	NI ^††^
*gluQUO: Control Your Diabetes*	Inglês	Ferramenta para gerenciar a DM ^*^	2.3	NI ^††^	NI ^††^
*Happy Bob*	Inglês	Ferramenta com conselhos encorajadores para o controle da DM ^*^	1.9	NI ^††^	NI ^††^
*Health2Sync*	Inglês	Diário da glicemia	2.6	NI ^††^	NI ^††^
*Help Diabetes*	Inglês	Ferramenta para realizar a contagem de carboidratos	3.1	NI ^††^	NI ^††^
iGlicho	Português	Gerenciar a DM ^*^	0.8	NI ^††^	NI ^††^
*Insulin Calculator*	Português	Realizar o cálculo de doses de insulina	1.0	NI ^††^	NI ^††^
Os pacientes diabéticos, acompanhar e monitorar seus níveis de glicose no sangue	Português	Gerenciar a DM ^*^	1.6	NI ^††^	NI ^††^
*Undermyfork: Diabetes*	Inglês	Diário de alimentação para os indivíduos com DM ^*^	2.12	NI ^††^	NI ^††^

^‡^ BD = *Becton Dickinson* ;

^¶^ BG = *Blood Glucose* ;

^**^ BP = *Blood Pressure* ;

^||^ HbA1c = Hemoglobina glicada;

^§^ PA = Pressão Arterial;

^†^ *App* = Aplicativo;

^*^ DM = *Diabetes Mellitus* ;

^††^ NI = Não Identificado

 A Figura 3 apresenta a categorização dos *apps* analisados quanto aos eixos temáticos e os temas abordados. Os temas predominantes foram manejo da glicemia (55; 77,4%), seguido de alimentação (41; 57,7%), terapia medicamentosa oral e insulinoterapia (39; 54,9%). Alguns aplicativos contemplam mais de um tema em seu conteúdo, o que permite que se enquadrem em uma ou mais categorias concomitantemente. 

**Figura 3:** - Categorização dos *apps* quanto aos eixos temáticos e temas abordados. Macaé, RJ, 2023

 Em relação aos recursos disponíveis nos *apps* , foram identificados: diários (50; 70,4%); gráficos (46; 64,7%); figuras/imagens (37; 52,1%); relatórios (27; 38%); lembretes/alarmes (17; 23,9%), estatísticas (7; 9,8%) e tabelas (5; 7%). Também foram identificados recursos relacionados aos hábitos de vida e bem-estar: registro da pressão arterial (22; 30,9%), peso (14; 19,7%), monitoramento de atividades físicas (15; 21,1%) e orientações/metas (2; 2,8%) que auxiliam na gestão eficaz da DM. Em alguns *apps* era permitida a inserção de dados sobre o perfil sociodemográfico do usuário (47; 66,2%), e clínico (52; 73,2%), como o ano do diagnóstico (9;12,6%) e o tipo de DM (24; 33,8%). 

 No que tange à usabilidade dos *apps* analisados, dois (2,8%) obtiveram um escore ≥ 85; 34 (47,9%) pontuaram entre 68-84 pontos; 29 (40,8%) apresentaram escores entre 50-67 pontos; seis (8,5%) obtiveram escores inferiores a 50. A Figura 4 ilustra as informações dos *apps* sobre o conteúdo, os recursos e usabilidade. 

**Figura 4:** - Caracterização dos aplicativos selecionados quanto ao conteúdo, recursos e usabilidade. Macaé, RJ, 2023

Aplicativo	Conteúdo	Recursos	Usabilidade (pontos no SUS ^*^ )
Usabilidade excelente (n=2)			
*forDiabetes* : Aplicativo de diabetes	Glicemia, medicações, PA ^†^ , refeições, atividade física, peso, HbA1c ^‡^ e artigos sobre a DM ^§^ .	Textos, figuras, gráficos, lembretes, compartilhamento de relatórios, sincronização com outros dispositivos; inserir as atividades diárias, refeições, medicações, níveis de glicemia, PA ^†^ , HbA1c ^‡^ e peso.	85,0
*Carbok: Carb Couting & Management for Diabetic*	Alimentação e contagem de carboidratos.	Figuras, textos, calculadora, gráficos; permite registrar as características das refeições.	85,0
Usabilidade aceitável (n=37)			
*mySugr*	Diário da glicemia, gerenciamento de medicamentos, insulina e ingestão de carboidratos.	Textos, gráficos e figuras. Permite adicionar lembretes, registrar glicemia, passos/atividade física, insulina/medicações, peso e enviar relatórios.	82,5
*Diabetes: Management & Blood Sugar Tracker App* ^||^	Registro da glicemia, gerenciamento de medicamentos, alimentos, peso, PA ^†^ , atividade física e exames laboratoriais.	Textos, gráficos e figuras. Possibilita registros de glicemia, alimentos e medicamentos, definição de alarmes, além de acompanhar a evolução por meio de gráficos e de relatórios.	82,5
A minha glycemia	Glicemia capilar, insulina, HbA1c ^‡^ e PA ^†^ .	Inserir valores de glicemia, lembretes, tabelas, histogramas, bloco de notas e adicionar contatos.	82,5
*Blood Glucose Tracker*	Alimentação, peso, PA ^†^ , glicemia e medicamentos.	Textos, gráficos, figuras e blocos de notas.	82,5
*Center Health – The Diabetes App* ^||^	Diário da glicemia, insulina, contagem de carboidratos, exercícios e sono.	Textos, gráficos, figuras, registro de dados sincronização com sensores, importação e exportação de planilhas.	82,5
Contagem de Carboidratos	Contagem de carboidratos.	Gráficos, textos e calculadora.	82,5
Glic Diabetes e Glicemia	Glicemia, insulina, PA ^†^ , medicamentos e artigos educacionais.	Figuras, textos, gráficos, alerta de hipoglicemia, tabela nutricional, cálculo de insulina, contagem de carboidratos, conecta o prontuário eletrônico e envia o relatório dos dados.	82,5
*iGlicho*	Alimentação, diário de glicemia, contagem de carboidratos e insulina.	Gráficos, textos, tabela de alimentos, contagem de carboidratos, cálculo de insulina e geração de relatórios.	82,5
Controle de Glicose	Glicemia, HbA1c ^‡^ , insulina, medicamentos, PA ^†^ e alimentação.	Gráficos, textos. Possibilita adicionar exames laboratoriais, visualizar os níveis de glicose e PA ^†^ em gráficos e exportar os relatórios.	80,0
*Smart Diabetes Diary*	Glicemia, insulina e medicamentos.	Figuras, gráficos, estatísticas, textos, bloco de notas e exportação de dados.	80,0
*Diabetes Logbook – Blood Glucose Tracker*	Diário da glicemia, HbA1c ^‡^ , contagem de carboidratos e insulina.	Textos, gráficos e figuras.	80,0
Diabética Comida Receitas *App* ^||^	Alimentação.	Textos e imagens.	80,0
*Health2Sync*	Diário da glicemia, alimentação, contagem de carboidratos, atividade física, PA ^†^ e peso.	Gráficos, textos, figuras. Permite adicionar fotos das refeições e gerar relatórios.	80,0
*Dits*	Glicemia capilar e artigos educacionais.	Gráficos, textos, figuras e artigos.	80,0
*Blood Sugar Log – Diabetes Tracker*	PA ^†^ , glicemia, Hb1Ac ^‡^ , peso e medicamentos.	Figuras, textos e envio de relatórios.	77,5
*Diabetes Meal Plan*	Alimentação.	Imagens, textos e calculadora.	77,5
*Blood Glucose Tracker*	Glicemia, PA ^†^ , medicamentos, Hb1Ac ^‡^ e peso.	Gráficos, estatísticas, textos e bloco de notas.	77,5
*Diabetes Tracker App* ^||^ : *Blood Glucose & Cholesterol*	PA ^†^ , peso, glicemia, HbA1c ^‡^ e colesterol.	Textos, lembretes e gráficos.	77,5
*Diabetes Tracker*	Atividade física, alimentação, medicamentos, PA ^†^ e glicemia.	Figuras, lembretes, pesquisa por voz, adicionar lista de profissionais, data de consultas e de relatórios.	77,5
Glicose Companheiro	Glicemia e peso.	Gráficos, estatísticas, textos, calculadora e relatórios.	77,5
*Happy Bob*	Automonitorização glicêmica, metas e conselhos para controle da DM ^§^ .	Textos, figuras.	77,5
*Blood Sugar Diary for Diabetes*	Automonitorização glicêmica.	Textos, estatísticas, notificações, gráficos, alteração do tema, artigos e sincronização com os sensores.	77,5
SocialDiabetes	Sinais vitais, diário da glicemia, HbA1c ^‡^ , medicamentos, alimentação, atividade física, encorajamento e conteúdos educacionais.	Textos, calculadora, estimativa da HbA1c ^‡^ , alarmes, sincronização com os sensores e exportação de relatórios.	75,0
*Diabetes Health Manager*	Sintomas, medicamentos, tratamento e monitorização glicêmica.	Figuras, textos, gráficos e artigos.	75,0
Diabéticos Receitas	Receitas saudáveis.	Textos e figuras. Permite favoritar receitas, criar listas de compras e buscar receitas por meio da seleção de um ingrediente/alimento específico.	72,5
*Blue Circle Diabetes*	Atividade física, alimentação, insulina e glicemia.	Textos, imagens, calculadora, loja virtual, informações nutricionais, receitas e encontros on-line sobre a DM ^§^ .	72,5
*Eglucomonitor – Sugar & Diabetes Monitor App* ^||^	Diário da glicemia e insulina.	Textos e gráficos.	72,5
*Diabetes Diary*	Glicemia, contagem de carboidratos e insulina.	Gráficos, textos e calculadora.	72,5
*BD* ^¶^ *Diabetes Care*	Registro da glicemia e insulina; receitas saudáveis, perguntas e respostas sobre a DM ^§^ e os conteúdos educacionais.	Textos e figuras. Possibilita o registro de glicemia, insulina, compartilhamento de relatórios, lembretes, conexão com os outros dispositivos, perguntas e respostas, pesquisar receitas e conteúdos educacionais e definir metas diárias.	70,0
*Diabetes Diary – Blood Glucose Tracker*	PA ^†^ , peso, HbA1c ^‡^ , medicamentos e glicemia.	Textos, gráficos e relatórios.	70,0
*Intellin Diabetes Log Tracker & Risk Manager App*	Atividade física, PA ^†^ , glicemia e insulina.	Textos, figuras, gráficos, sincronização com os outros dispositivos e o compartilhamento de relatórios.	70,0
*Glycemic Index*	Alimentação.	Textos e ferramentas para adicionar novos alimentos.	70,0
*Undermyfork: Diabetes*	Alimentação e diário glicêmico.	Textos, imagens e gráficos.	70,0
*Insulin Calculator*	Insulina.	Textos e gráficos. Permite criar refeições, calcular insulina e obter histórico.	70,0
*BG* ^**^ *Monitor Diabetes*	Diário da glicemia, insulina, atividade física e alimentação.	Gráficos e textos. Possui lembretes e relatórios.	67,5
*Diabetes Tracker* Gratuito	Diário glicêmico.	Textos, gráficos e lembretes.	67,5
Receitas para Diabéticos	Alimentação.	Imagens e textos.	67,5
Usabilidade limítrofe (n=26)			
*Diabetes Control App* ^||^	Alimentação, insulina, medicamentos e glicose sanguínea.	Textos e gráficos. Lembrete, bloco de notas, adicionar contato de profissionais, *chat* e relatórios.	65,0
Diabetes Controle	Automonitorização glicêmica.	Gráficos, estatísticas, figuras, textos e compartilhar relatórios.	65,0
*Blood Glucose Tracker – Track Your Blood Glucose*	Diário glicêmico, PA ^†^ e peso.	Textos, gráficos e exportar os dados.	65,0
Glucômetro – Rastreamento de Diabetes	Diário da glicemia, insulina e HbA1c ^‡^ .	Inserir os níveis de glicose, HbA1c ^‡^ e insulina.	65,0
*Diabetes Test My Blood Sugar Diary – BP* ^††^ *Glucose*	Automonitorização glicêmica, PA ^†^ , peso, HbA1c ^‡^ e medicações.	Figuras, textos, gráficos e exportar relatórios.	65,0
*Blood Sugar Diary - Blood Glucose Tracker*	Alimentação, medicamentos e glicemia.	Textos, figuras, gráficos e artigos sobre a DM ^§^ .	65,0
*GlucoseMonitor*	Diário glicêmico e sinais vitais.	Gráficos, textos e lembretes.	65,0
*gluQUO: Control Your Diabetes*	Alimentação, atividade física e diário da glicemia.	Textos, figuras, gráficos, lembretes, calculadora; permite adicionar fotos das refeições e obter relatórios para compartilhar.	65,0
*GlicAPP*	Exames laboratoriais, glicemia e insulina.	Textos. Possibilita o registro de dados médicos, exames laboratoriais e sugestões.	65,0
*My Sugar Diary – Diabetes App* ^||^	Diário glicêmico.	Textos, tabelas e figuras.	62,5
*Diabetes Pro*	Diário da glicemia e medicamentos.	Figuras, textos, gráficos e ferramenta para realizar rodízio de dados para verificação da glicemia capilar.	62,5
*Diabetes Food Recipes*	Alimentação.	Textos e imagens.	60,0
*Diabetes Pilot Pro*	Diário glicêmico, alimentação e contagem de carboidratos.	Textos e gráficos.	60,0
Os pacientes diabéticos, acompanhar e monitorar seus níveis de glicose no sangue	Diário da glicemia.	Textos, tabelas, gráficos e lembretes.	60,0
Diário Diabetes	Atividade física, alimentação, PA ^†^ , peso, glicose, HbA1c ^‡^ e medicamentos.	Textos, gráficos, médias dos dados inseridos e lembretes.	57,5
*Glucool Diabetes*	Glicemia, insulina, PA ^†^ , contagem de carboidratos, HbA1c ^‡^ , exercícios e medicações.	Gráficos, textos, calculadora e exportação de dados.	57,5
*Joddi – Diabetes LogBook*	Diário da glicemia e insulina.	Textos, registrar e exportar dados.	57,5
*Diabetic Diet Plan* (v. 1.0)	Alimentação.	Textos e imagens.	57,5
*Diabetic Diet Plan* (v. 1.3)	Alimentação.	Imagens e textos.	57,5
*glucoSecrets*	Diário da glicemia, atividade física e medicamentos.	Gráficos/estatísticas, textos e figuras.	57,5
*Help Diabetes*	Contagem de carboidratos.	Textos e calculadora.	57,5
*Beat Diabetes*	Alimentação.	Imagens e textos.	52,5
Diabete – Diário Glucose	Glicemia e peso.	Gráficos, estatísticas, lembretes, inserir os valores, exportar e importar os dados.	52,5
*Wecheck – Diabetes Lifelog*	Sinais vitais, alimentação, insulina e glicemia.	Gráficos, textos, registro de sinais vitais, glicose e insulina; conexão NFC ^‡‡^ , *Bluetooth* e sincronização com os aparelhos *Wecheck* .	52,5
*Butterfly – Diabetes App* ^||^	Metas semanais para controle da DM ^§^ .	Textos e registro de dados.	52,5
*Diabetes SmartManager*	Contagem de carboidratos, insulina e diário da glicemia.	Textos, lembretes, gráficos. Permite registrar glicemia e insulina, modificar cor do tema e obter os relatórios.	50,0
Usabilidade inadequada (n=6)			
Diário de Sangue	Diário glicêmico, PA ^†^ e peso.	Gráficos, calendário e registro de glicemia, PA ^†^ e peso.	47,5
*Blood Sugar Diary*	Diário da glicemia.	Textos e gráficos.	45,0
*Diabetic Recipes: Great Recipes for Diabetics*	Alimentação.	Figuras, textos e áudios.	45,0
*DiabetesDocs*	Atividade física, alimentação, PA ^†^ , glicemia e insulina.	Textos, gráficos e bloco de notas.	40,0
Diabetes Ginástica	Atividade física.	Textos, imagens e vídeos explicativos dos exercícios.	37,5
*Sukar – Blood Glucose & Diabetes Tracker App* ^||^	Ingestão hídrica, glicemia, contagem de carboidratos, HbA1c ^‡^ , peso e medicações.	Textos, figuras e compartilhamento de relatórios.	25,0

^*^ SUS = *System Usability Scale* ;

^¶^ BD = *Becton Dickinson* ;

^**^ BG = *Blood Glucose* ;

^††^ BP = *Blood Pressure* ;

^‡‡^ NFC = *Near Field Communication*

^§^ DM = *Diabetes Mellitus* ;

^†^ PA = Pressão Arterial;

^||^ *App* = Aplicativo;

^‡^ HbA1c = Hemoglobina glicada;

## Discussão

A interpretação dos dados mais abrangentes indica a predominância de aplicativos orientados para a gestão da glicemia, da terapia medicamentosa e de uma categorização de usabilidade aceitável, seguida da limítrofe. Disso, infere-se existir a necessidade de um investimento no aprimoramento destes aplicativos para garantir que o usuário possa utilizar melhor o recurso, ampliando suas possibilidades de suporte. Ademais, a predominância da visão focada na glicemia e no controle farmacológico pode enfatizar a visão biologicista e centrada nos profissionais, minimizando as alternativas de papel mais ativo da pessoa, especialmente relacionadas aos comportamentos e às crenças afeitas aos conceitos mais ampliados de saúde.

 Entretanto, os conteúdos e os recursos encontrados nos “aplicativos para a gestão da glicemia e da terapia medicamentosa”, “aplicativos para as mudanças no estilo de vida”, e “aplicativos para as medidas de bem-estar e educação” possuem, de forma complementar, implicações diretas nas atitudes para o autocuidado. Isto porque as atitudes de autocuidado são determinadas por questões comportamentais, cognitivas e emocionais ^(^ ^16^ ^-^ ^17^ ^)^ e estes conteúdos e recursos facilitam o desenvolvimento de comportamentos de saúde positivos por incluir lembretes/alarmes, diários de monitoramento, relatórios e gráficos. Utilizam atitudes cognitivas baseadas no acesso às orientações e nas metas de saúde, que reforçam as respostas emocionais positivas por meio de conselhos motivacionais e recompensadores dos bons resultados alcançados. Assim, a pessoa com DM pode ser orientada a utilizar mais de um aplicativo dos diferentes eixos, de forma a explorar as características complementares. 

 Em relação aos recursos dos *apps* classificados no eixo “Aplicativos para a gestão da glicemia e da terapia medicamentosa” englobaram o apoio ao manejo das variações da glicemia e a utilização dos medicamentos. Algumas pesquisas investigam o impacto destes recursos no enfrentamento aos desafios de alcançar as metas glicêmicas individuais, na tomada de decisão frente aos padrões da glicemia e no uso dos medicamentos. Uma revisão sistemática que avaliou o efeito dos *apps* para *smartphones* no controle glicêmico em pacientes jovens com DM Tipo 1 (DM1) não revelou uma redução significativa na Hemoglobina Glicada (HbA1c), em comparação aos cuidados habituais em combinação. No entanto, identificaram-se que *apps* auxiliares com as calculadoras de insulina ou carboidratos foram benéficos na redução da HbA1c ^(^ ^18^ ^)^ . 

 Em paralelo, uma investigação do tipo mundo real indicou que o uso contínuo de *apps* de gerenciamento da glicemia está associado ao alcance de metas glicêmicas na prática clínica ^(^ ^19^ ^)^ . Já em participantes com DM2, os usuários que fizeram *login* em um *app* por 19 dias e tiveram pelo menos 85 dias desde o primeiro até o último *login,* tiveram aumento de mais de duas vezes a chance de reduzir o valor absoluto de HbA1c em 0,5% ^(^ ^20^ ^)^ . Parte destas contribuições pode estar associada às diferentes funções encontradas nos *apps* , razão pela qual importa considerar a funcionalidade dos *apps,* para que haja melhores possibilidades de utilização destes pelos pacientes. Depreende-se desse processo o entendimento de que a qualidade da dinâmica no uso de *apps* para a saúde sofre a influência significativa da funcionalidade dessas tecnologias ^(^ ^21^ ^)^ . 

 Em relação aos conteúdos, alguns *apps* foram além da monitorização glicêmica usual, com a utilização de lembretes, diários, estatísticas, gráficos e relatórios do usuário, sendo possível fazer ajustes adequados e tomadas de decisão mais acuradas, dada a abrangência das informações obtidas, e, assim, melhorar a funcionalidade dos *apps* para potencializar sua eficácia e efetividade. 

 No que tange aos recursos que auxiliam no tratamento medicamentoso, destacam-se as ferramentas que buscam auxiliar na adesão medicamentosa. Ainda não estão evidentes na literatura as contribuições destes recursos para a adesão ao tratamento. A avaliação de *apps* de alta qualidade disponíveis gratuitamente ao público mostrou que quase metade destes não promoveram alta ou moderada adesão medicamentosa. No entanto, destaca-se a limitação imposta pela amostra reduzida e a falta de padronização dos instrumentos avaliativos sobre a adesão medicamentosa ^(^ ^22^ ^)^ . 

 O eixo “Aplicativos para a mudança no estilo de vida” contemplou *apps* com conteúdos e recursos voltados à prática de atividades físicas, alimentação, controle pressórico e de peso. A identificação de *apps* que contemplam este eixo se coaduna com uma investigação sobre o uso de dispositivos móveis por pacientes que convivem com DM, a qual evidenciou que uma parcela significativa dos participantes utiliza *app* para o planejamento nutricional e dietético (85,5%), monitorização do controle glicêmico (76,5%) e agendamento de consultas de saúde (90,5%) ^(^ ^23^ ^)^ . 

 Adicionado a estas evidências, uma revisão sistemática sobre a efetividade de *apps* móveis relacionados ao estilo de vida de pessoas com DM detectou que o gerenciamento dos hábitos de vida por meio de *apps* pode auxiliar a alcançar, a curto prazo, o controle glicêmico efetivo. No entanto, não se observaram contribuições significativas no controle da pressão arterial e do peso ^(^ ^7^ ^)^ . 

 O eixo “Aplicativos para as medidas de bem-estar e educação” abrangeu ferramentas que auxiliavam no estabelecimento de metas, orientações educativas, conselhos motivacionais e encorajadores frente ao convívio com a cronicidade da DM. Destaca-se a capacidade da motivação ser um importante para que as pessoas com DM consigam lidar de maneira eficaz com a doença ^(^ ^24^ ^)^ . Notavelmente, tais aspectos são observados nos *apps* analisados, incluindo a temática educacional, que também se caracteriza como relevante para a manutenção da saúde de pessoas com DM. Nesse contexto, os *apps* parecem emergir como uma ferramenta capaz de fornecer orientações educativas e informações relevantes para os indivíduos de forma instantânea, contribuindo com o seu processo de ensino-aprendizagem e o manejo do seu quadro de saúde ^(^ ^8^ ^)^ . 

 Apesar de os aspectos motivacionais terem sido objetivos de alguns *apps* , não foram identificados conteúdos voltados à saúde mental. Sabe-se que pessoas com DM são mais suscetíveis à depressão, à ansiedade, ao isolamento social e aos transtornos alimentares ^(^ ^25^ ^)^ . Além do impacto que o tratamento causa, como estresse físico e emocional na pessoa e na família, a convivência com essa condição crônica é permeada por inúmeros desafios, os quais incluem a adaptação a uma nova rotina de cuidados ^(^ ^4^ ^,^ ^25^ ^-^ ^26^ ^)^ . 

 Aponta-se, também, que outras temáticas importantes para a saúde de pessoas com DM não foram abordadas pelos *apps* analisados, como a sexualidade e reprodução. A sexualidade é um componente essencial para a manutenção de uma vida saudável, e repercussões biológicas, psicológicas e sociais podem sofrer influência da DM ^(^ ^27^ ^)^ . Outras temáticas pouco exploradas foram as orientações educativas sobre as complicações da DM, como a neuropatia diabética e a Doença Arterial Periférica. As informações relativas a essas complicações e à prevenção de lesões cutâneas são fundamentais para o manejo da saúde, contribuindo para a redução dos custos em saúde e evitando internações hospitalares por causas preveníveis, o que pode resultar em maior qualidade de vida ^(^ ^28^ ^)^ . 

 Outro aspecto importante é a inexistência de aplicativos voltados à pessoa com deficiência. Não foram identificados conteúdos relacionados à deficiência visual ou baixa acuidade visual decorrentes de outra complicação comum da DM, como a retinopatia diabética. A ausência de aplicativos sobre o tema é alarmante, uma vez que já são reconhecidas as contribuições dos avanços tecnológicos para a inclusão da pessoa com deficiência na sociedade, minimizando as desigualdades, dirimindo barreiras, estimulando a autonomia, o autocuidado e garantindo maior acessibilidade ^(^ ^29^ ^)^ . Assim, se reconhece a necessidade de investimentos no desenvolvimento de ferramentas para essas temáticas. 

 Apesar do exposto, cumpre-se destacar que em meio à pluralidade de 23 países desenvolvedores dos *apps* selecionados, apenas dois são ibero-americanos (Brasil e Espanha), cuja expressão total do número de *apps* não ultrapassou 11 (2%). Essa realidade pode sinalizar um importante indicador de expressão em investimento tecnológico relacionado à saúde da pessoa com DM, sobretudo, por corresponder às realidades com características socioculturais distintas, cujos reflexos podem sugerir impactos distintos em populações mais vulneráveis em que a alfabetização funcional, cultural, tecnológica e o letramento imprimem realidades diferentes entre os povos e as nações ^(^ ^30^ ^-^ ^31^ ^)^ , o que não é diferente para as pessoas com DM. 

 Ademais, do total de 71 *apps* selecionados, apenas 16 apresentam versão em português e um em espanhol. Desses *apps* , nenhum foi caracterizado com excelente aceitação de usabilidade, sendo a maioria considerada de usabilidade aceitável (58,8%). Frente a isso, evidencia-se a necessidade de se ter mais e melhores discussões no âmbito ibero-americano sobre as tecnologias para a saúde das pessoas com DM, com vistas a aproximar as informações sobre o manejo da glicemia, a terapia medicamentosa, as mudanças do estilo de vida e as medidas de bem-estar da realidade plural e objetiva dessa população. 

 Em uma perspectiva técnica, constata-se que as versões dos *apps* analisados eram crescentes, indicando a atualização e o aprimoramento dos conteúdos, apontando adição de funcionalidades. Observou-se, ainda, que o número de *downloads* era muito variado, o que equivale ao número de pessoas que acessa a loja virtual e realiza o *download* . Contudo, esse número pode não refletir o número real de usuários dos *apps* , pois apenas baixá-los não torna o indivíduo automaticamente usuário dos conteúdos e recursos do aplicativo. 

 Cabe destacar que, embora a expressiva maioria dos *apps* tenha apresentado a versão em inglês, com 53/71, o número de *downloads* , quando comparado aos *apps* de língua portuguesa, de apenas 16/71, não foi tão superior quanto a diferença entre essas duas modalidades. Para os *apps* em língua inglesa, o número total de *downloads* foi superior a 2.217.500, enquanto que os *apps* em português somaram mais de 1.960.100 *downloads* . Essa realidade pode revelar comportamentos diferentes entre grupos sociais de culturas distintas, sobre a importância que atribuem às práticas de saúde e/ou da utilização de tecnologias para diferentes finalidades, o que inclui acessar as informações e estabelecer as rotinas relacionadas à saúde. Nesse sentido, o Brasil, por exemplo, é o país cujos usuários demandam mais tempo de uso diário de *smartphone* ( *Android* ), com 5,4 horas/dia, conforme revelou o relatório da plataforma *AppAnnie* , de 2021 ^(^ ^32^ ^)^ . 

 Neste estudo verificou-se que a maioria dos *apps* apresentava usabilidade aceitável ou os recursos apresentavam algumas falhas, mas com padrões de usabilidade ainda aceitáveis. Tais resultados corroboraram os dados de uma revisão sistemática que identificou os índices de satisfação de especialistas e pacientes entre 38% a 80% ^(^ ^12^ ^)^ . Os principais problemas de usabilidade são tarefas de várias etapas, funcionalidade e interação limitadas e navegação difícil no sistema. Outro estudo, que avaliou os aplicativos móveis gratuitos em espanhol, identificou poucos aplicativos gratuitos para o controle da DM disponíveis, sendo a maioria sem certificação de qualidade e poucos fornecendo referências científicas sobre seu conteúdo ^(^ ^13^ ^)^ . 

 Esta pesquisa fornece um panorama abrangente sobre os *apps* móveis voltados à saúde de pessoas com DM disponíveis nas principais plataformas, bem como uma análise sobre os seus recursos, conteúdos e usabilidade. Este panorama identificou as limitações dos recursos e os temas que ainda requerem desenvolvimento. Frente aos resultados, profissionais e pesquisadores da Enfermagem e da área da saúde podem extrair elementos que facilitam tanto a avaliação detalhada dos *apps* , quanto à recomendação mais segura daqueles que atenderiam melhor a determinados critérios, bem como direcionar o investimento no desenvolvimento de novas tecnologias e recursos móveis necessários ao manejo da saúde de pessoas com DM. 

 Como limitação deste estudo, menciona-se a não inclusão dos *apps* pagos, que exigiam recursos específicos ou autorização dos seus desenvolvedores. Como o estudo centrou-se nos aspectos da usabilidade, existe a limitação da confiabilidade do conteúdo empregado pelos desenvolvedores e a observância dos critérios de certificação de qualidade, o que exige estudos futuros e uma apreciação destes parâmetros pelos profissionais de saúde que forem recomendar os *apps* . 

## Conclusão

 Os *apps* móveis voltados à saúde de pessoas com DM disponíveis nos principais sistemas operacionais possuem conteúdos e recursos variados. Apresentam, em sua maioria, usabilidade aceitável e envolvem principalmente as temáticas do manejo da glicemia, alimentação, terapia medicamentosa e insulinoterapia. Têm potencial para auxiliar e orientar os usuários no manejo da doença cotidianamente, além de se constituírem como uma possível estratégia de cuidado a ser utilizada pelos profissionais de saúde. 

 No entanto, algumas temáticas foram pouco contempladas, como a saúde mental, sexual e reprodutiva, as quais podem contribuir para o plano terapêutico em uma perspectiva holística e integral. Evidenciou-se também a necessidade de desenvolvimento de *apps* mais inclusivos, com conteúdos e recursos voltados às pessoas com DM que convivem com algum tipo de deficiência. Por fim, a recomendação de uso pelos profissionais deve ser antecedida pela verificação da confiabilidade das informações contidas nos *apps* , questão que, pela finalidade desta pesquisa, não foi avaliada.
